# Mild intermittent hypoxia exposure induces metabolic and molecular adaptations in men with obesity

**DOI:** 10.1016/j.molmet.2021.101287

**Published:** 2021-07-03

**Authors:** Rens L.J. van Meijel, Max A.A. Vogel, Johan W.E. Jocken, Lars M.M. Vliex, Joey S.J. Smeets, Nicole Hoebers, Joris Hoeks, Yvonne Essers, Paul F.M. Schoffelen, Henrike Sell, Sander Kersten, Kasper M.A. Rouschop, Ellen E. Blaak, Gijs H. Goossens

**Affiliations:** 1 Department of Human Biology, NUTRIM School of Nutrition and Translational Research in Metabolism, Maastricht University Medical Center^+^, Maastricht, the Netherlands; 2 Department of Nutrition and Movement Sciences, NUTRIM School of Nutrition and Translational Research in Metabolism, Maastricht University Medical Center^+^, Maastricht, the Netherlands; 3 Paul-Langerhans-Group for Integrative Physiology, German Diabetes Center, Dusseldorf, Germany; 4 Nutrition, Metabolism and Genomics Group, Division of Human Nutrition and Health, Wageningen University, Wageningen, the Netherlands; 5 Maastricht Radiation Oncology (MaastRO) Laboratory, GROW-School for Oncology and Developmental Biology, Maastricht University Medical Center^+^, Maastricht, the Netherlands

**Keywords:** Hypoxia exposure, Obesity, Insulin sensitivity, Substrate metabolism, Inflammation, RCT

## Abstract

**Objective:**

Recent studies suggest that hypoxia exposure may improve glucose homeostasis, but well-controlled human studies are lacking. We hypothesized that mild intermittent hypoxia (MIH) exposure decreases tissue oxygen partial pressure (pO_2_) and induces metabolic improvements in people who are overweight/obese.

**Methods:**

In a randomized, controlled, single-blind crossover study, 12 men who were overweight/obese were exposed to MIH (15 % O_2_, 3 × 2 h/day) or normoxia (21 % O_2_) for 7 consecutive days. Adipose tissue (AT) and skeletal muscle (SM) pO_2_, fasting/postprandial substrate metabolism, tissue-specific insulin sensitivity, SM oxidative capacity, and AT and SM gene/protein expression were determined. Furthermore, primary human myotubes and adipocytes were exposed to oxygen levels mimicking the hypoxic and normoxic AT and SM microenvironments.

**Results:**

MIH decreased systemic oxygen saturation (92.0 ± 0.5 % vs 97.1 ± 0.3, *p* < 0.001, respectively), AT pO_2_ (21.0 ± 2.3 vs 36.5 ± 1.5 mmHg, *p* < 0.001, respectively), and SM pO_2_ (9.5 ± 2.2 vs 15.4 ± 2.4 mmHg*, p* = 0.002, respectively) compared to normoxia. In addition, MIH increased glycolytic metabolism compared to normoxia, reflected by enhanced fasting and postprandial carbohydrate oxidation (*p*_AUC_ = 0.002) and elevated plasma lactate concentrations (*p*_AUC_ = 0.005). Mechanistically, hypoxia exposure increased insulin-independent glucose uptake compared to standard laboratory conditions (~50 %, *p* < 0.001) and physiological normoxia (~25 %, *p* = 0.019) through AMP-activated protein kinase in primary human myotubes but not in primary human adipocytes. MIH upregulated inflammatory/metabolic pathways and downregulated extracellular matrix-related pathways in AT but did not alter systemic inflammatory markers and SM oxidative capacity. MIH exposure did not induce significant alterations in AT (p = 0.120), hepatic (p = 0.132) and SM (p = 0.722) insulin sensitivity.

**Conclusions:**

Our findings demonstrate for the first time that 7-day MIH reduces AT and SM pO_2_, evokes a shift toward glycolytic metabolism, and induces adaptations in AT and SM but does not induce alterations in tissue-specific insulin sensitivity in men who are overweight/obese. Future studies are needed to investigate further whether oxygen signaling is a promising target to mitigate metabolic complications in obesity.

**Clinical trial registration:**

This study is registered at the Netherlands Trial Register (NL7120/NTR7325).

## Highlights

• MIH exposure decreases oxygen partial pressure in human adipose tissue and skeletal muscle.
• MIH exposure induces a shift in substrate utilization toward glycolytic metabolism.
• Hypoxia exposure increases insulin-independent glucose uptake in primary human myotubes, at least in part through AMPK.
• MIH does not alter adipose tissue, hepatic and peripheral insulin sensitivity.

## 1 Introduction

Oxygen homeostasis, or the balance between oxygen supply and demand, is critical in cellular survival. Indeed, alterations in tissue oxygen partial pressure (pO_2_) impact a variety of physiological responses. Compelling evidence points toward the important role of altered oxygen availability (i.e., ‘*hypoxia*’) in cardiometabolic perturbations [1,2]. Intriguingly, although metabolic adaptations to hypoxia are not fully understood, oxygen availability in crucial metabolic organs such as adipose tissue (AT) and skeletal muscle (SM) may play a significant role in the pathophysiology of obesity-related cardiometabolic complications [2].

We have recently demonstrated that AT pO_2_ was increased in obese insulin resistant individuals compared to lean and obese insulin-sensitive individuals [3,4]. Moreover, AT pO_2_ was positively associated with insulin resistance, independently of age, sex, and adiposity [3]. In line with these findings, diet-induced weight loss markedly decreased AT pO_2_ in humans, accompanied by improved insulin sensitivity [5]. These findings suggest that targeting tissue oxygenation may lower the risk for cardiometabolic complications in people with obesity [2,6].

Conflicting findings on the effects of pO_2_ on inflammation and insulin sensitivity have also been reported, which may be related to the severity, pattern, and duration of hypoxia exposure [2]. Indeed, acute exposure to severe hypoxia impaired insulin signaling in both murine and human adipocytes [7], whereas the prolonged, repeated exposure of differentiating adipocytes to transient hypoxia was able to reprogram these cells for enhanced insulin sensitivity [8]. Furthermore, hypoxia exposure increased glucose uptake in murine and human myotubes and human SM explants [9,10]. Interestingly, prolonged mild intermittent hypoxia (MIH) exposure, characterized by multiple hypoxic episodes per day, has been shown to improve glucose homeostasis in rodents [[11], [12], [13], [14]]. Moreover, an uncontrolled trial in obese men indicated that mild hypoxia exposure (15 % O_2_) improved whole-body insulin sensitivity [15]. However, placebo-controlled trials investigating the effects of prolonged exposure to MIH on tissue-specific insulin sensitivity and cardiometabolic risk factors in humans are lacking. In addition, a more detailed mechanistic understanding of the putative beneficial effects of MIH on glucose homeostasis is warranted.

Therefore, we hypothesized that MIH improves insulin sensitivity and induces a shift toward increased glucose utilization in individuals that are overweight and obese. Here, we report the first randomized, controlled, single-blind crossover trial designed to investigate the effects of MIH exposure (FiO_2_ 15 % O_2_, 3 times 2 h/day vs. 21 % O_2_) for 7 consecutive days on tissue-specific insulin sensitivity (primary study outcome), AT and SM pO_2,_ and fasting and postprandial energy/substrate metabolism in overweight/obese men. The reason we applied this MIH protocol was due to exposure to 15 % O_2_ improving insulin sensitivity in a previous uncontrolled human study [15], and an intermittent pattern of mild hypoxia exposure (3–15 hypoxic episodes/day) might elicit more beneficial effects on the cardiovascular risk profile [16] without inducing adverse effects. Furthermore, we determined the impact of MIH on the gene/protein expression in AT and SM and performed functional mechanistic experiments using human primary myotubes and adipocytes cultured under oxygen levels mimicking the AT and SM microenvironments in humans.

## 2 Methods

### 2.1 Human intervention study

#### 2.1.1 Study design

Twelve overweight/obese men (BMI ≥28 kg/m^2^, age 30–65 years) with HOMA_IR_ ≥2.2 participated in the present study. Exclusion criteria were smoking, cardiovascular disease, type 2 diabetes mellitus, liver or kidney malfunction, use of medication known to affect body weight, glucose and/or lipid metabolism, and marked alcohol consumption (>14 alcoholic units/wk). Furthermore, subjects had to be weight stable (weight change <3.0 kg) for at least three months prior to the start of the study.

Study participants were exposed to normobaric mild intermittent hypoxia (FiO_2_ 15 %; equivalent to ~2800 m above sea level) and normobaric normoxia (FiO_2_ 21 %) for 7 consecutive days (3 cycles of 2 h exposure/day, with 1 h of normoxia exposure between hypoxic cycles) in a randomized fashion and separated by a 3- to 6-week wash-out period (Supplementary Figure 1). A random allocation sequence was performed by an independent researcher using a computer-generated randomization plan (block size n = 4). Hypoxia exposure was performed in an in-house manufactured airtight clinical room with the capability to accurately adjust oxygen availability at the Metabolic Research Unit Maastricht (Maastricht University, The Netherlands). The oxygen level was set and maintained at 15.0 ± 0.2 % for the hypoxia exposure regimen. Participants were blinded for the exposure regimen (hypoxia or normoxia). Individuals were asked to refrain from drinking alcohol and perform no exercise 48 h before the start of and during exposure regimens. Furthermore, participants performed a standardized light-stepping activity (3 × 5 min, p. day, 15 steps/min) during days 1–7.

Measurements performed on days 6 (AT and SM pO_2_), day 7 (high-fat mixed-meal [HFMM] test), and day 8 (two-step hyperinsulinemic-euglycemic clamp), as explained below, were performed after an overnight fast of at least 10 h. Participants were kept under energy-balanced conditions. The diet was adjusted individually to match the energy requirements and maintain an energy balance throughout the study. Based on the estimated daily energy expenditure (basal metabolic rate [BMR; Ventilated Hood, Omnical, Maastricht University] multiplied by activity score of 1.55), subjects received a standardized diet consisting of 50 % carbohydrate, 35 % fat, and 15 % protein to maintain a stable body weight throughout the study.

An assessment of hunger and satiety was performed on days 1, 3, and 7 by visual analog scale (VAS) questionnaires. Adverse events of MIH were monitored by the Lake Louise questionnaire for Acute Mountain Sickness (AMS).

The study was approved by the Medical-Ethical Committee of Maastricht University and performed according to the Declaration of Helsinki. All subjects gave their written informed consent before participation in the study. This study is registered at the Netherlands Trial Register (NL7120/NTR7325).

#### 2.1.2 Anthropometric measurements

Body weight was measured to the nearest 0.1 kg (model 220, Seca). Height was measured using a wall-mounted stadiometer (Seca). Blood pressure and heart rate were assessed (UA-789XL digital blood pressure monitor, A&D medical) on multiple occasions during the exposure protocol (days 1–5).

#### 2.1.3 Systemic oxygen saturation, adipose tissue, and skeletal muscle partial oxygen pressure

Systemic oxygen saturation levels were continuously monitored throughout the exposure regimens by pulse oximetry (Nellcore N-595 Pulse oximeter, Nellcor).

On day 6 of each exposure regimen, AT and SM pO_2_ were measured by a highly accurate optochemical measurement system to continuously monitor tissue pO_2_ *in vivo* in humans (Figure 2A), as described previously [4]. Briefly, microdialysis catheters (CMA60, CMA microdialysis AB) were inserted into abdominal subcutaneous AT 6–8 cm lateral from the umbilicus (skin anesthetized using EMLA cream) and SM (*m. gastrocnemius*; local anesthesia using 2 % lidocaine after perfusion [Ringer solution]) After the insertion, both microdialysis catheters were perfused with Ringer solution (Baxter BV) at a flow rate of 2 μl/min (CMA400 microinfusion pump, CMA Microdialysis AB). The interstitial fluid was then directed toward a flow-through cell containing an optochemical O_2_-sensor [17]. Within 2–3 h after the insertion of the microdialysis probes in AT and SM, pO_2_ had reached stable values (change in pO_2_ < 2.0 mmHg over a 20-min period). Fasting AT and SM pO_2_ values were calculated by averaging the 20-min periods with stable pO_2_ readings.

#### 2.1.4 High-fat mixed-meal test

On day 7 of each exposure regimen, a high-fat mixed-meal challenge test was performed (Figure 2A), fasting and postprandial (0–240 min) blood samples were collected, and substrate oxidation was determined using indirect calorimetry. Following a 30-min baseline period under fasting conditions, individuals were asked to ingest a liquid test meal, providing 2.6 MJ (consisting of 61 E% fat [35.5 E% saturated fat, 18.8 E% monounsaturated fat, and 1.7 E% polyunsaturated fat], 33 E% carbohydrates, and 6.3 E% protein), at t = 0 within 5 min. Blood samples were collected from a superficial dorsal hand vein, which was arterialized by placing the hand into a hotbox (~55 °C). Blood samples were taken under fasting (t = 0 min) and postprandial conditions (t = 30, 60, 90, 120, 180, and 240 min). Energy expenditure and substrate oxidation were assessed using indirect calorimetry (open-circuit ventilated hood system, Omnical, Maastricht University) under fasting conditions (t = −30 - 0 min) and for 4 h after the ingestion of the high-fat mixed-meal [18]. Calculations of energy expenditure and substrate oxidation were performed according to the formulas of Weir [19] and Frayn [20]. Nitrogen excretion was based on the assumption that protein oxidation represents ~15 % of the total energy expenditure [21].

#### 2.1.5 Two-step hyperinsulinemic-euglycemic clamp

A two-step hyperinsulinemic–euglycemic clamp combined with a D-[6,6-^2^H_2_]-glucose tracer infusion (Cambridge Isotope Laboratories, no. DLM-349) was performed on day 8, under normoxic conditions (Figure 5A) to determine adipose tissue, hepatic, and peripheral insulin sensitivity [22]. First, a primed (bolus-injection of 2.4 mg∙kg^−1^) continuous infusion of D-[6,6-^2^H_2_]-glucose was started and continued throughout the measurement at 0.04 mg∙kg^−1^∙min^−1^ to determine the baseline endogenous glucose production (EGP), glucose rate of appearance (Ra), and glucose disposal (Rd). After 2 h, insulin was infused at a primed continuous low rate of 10 mU∙m^−2^∙min^−1^ for 3 h to assess hepatic insulin sensitivity (% suppression of EGP) and adipose tissue insulin sensitivity (% suppression of free fatty acids [FFA]), followed by insulin infusion at a high rate of 40 mU∙m^−2^∙min^−1^ for 2.5 h to determine peripheral insulin sensitivity (Rd). Arterialized blood samples (hotbox at ~55 °C) were frequently taken from a superficial dorsal hand vein, and blood glucose concentrations were directly determined. Euglycemia was maintained by a variable co-infusion of a 17.5 % glucose solution enriched by 1.1 % tracer (5.0 mmol∙l^−1^). During the last 30-min of each step (0, 10, and 40 mU∙m^−2^∙min^−1^ insulin), substrate oxidation was measured using indirect calorimetry, and blood was sampled every 15 min to determine steady-state kinetics.

#### 2.1.6 Skeletal muscle and adipose tissue biopsies

Before the start of the hyperinsulinemic-euglycemic clamp, an SM (80–100 mg; *m. vastus lateralis*) and abdominal subcutaneous AT biopsy (~1 g; 6–8 cm lateral from the umbilicus) were collected under local anesthesia (2 % lidocaine without epinephrine). Part of the fasting muscle biopsy was immediately placed in an ice-cold preservation medium (BIOPS, OROBOROS Instruments) to prepare intact permeabilized muscle fibers. Permeabilized muscle fibers were used to determine mitochondrial oxidative capacity using an oxygraph (OROBOROS Instruments), as described previously [23]. The remaining part of the SM biopsy was snap-frozen in liquid nitrogen and stored at −80 °C until the analysis. The AT specimen was washed using sterile saline, visible blood vessels were removed using sterile tweezers, and the AT biopsy was snap-frozen in liquid nitrogen and stored at −80 °C. Another muscle biopsy was collected at the end of the steady state of the high-insulin step of the clamp.

#### 2.1.7 Plasma biochemistry

Blood was collected into pre-chilled tubes, centrifuged at 1,000 *g*, and plasma was snap-frozen and stored at −80 °C until the analysis. Plasma glucose (ABX Pentra Glucose HK CP, Horiba ABX Diagnostics), FFA (WAKO NEFA-HR [2] ACS-ACOD method, WAKO Chemicals GmbH), TAG (ABX Pentra Triglycerides CP, Horiba ABX Diagnostics), and glycerol (Glycerol-kit UV-test, r-Biopharm) were determined. Plasma insulin was measured using a double-antibody radioimmunoassay (Millipore). Lactate was measured in plasma using standard enzymatic techniques automated on a Cobas Fara centrifugal spectrophotometer (Roche Diagnostics). Plasma TNFα, IFNγ, IL-6, and IL-8 concentrations were determined in fasting plasma samples collected at day 8, subsequent to 7-d MIH exposure, using the V-PLEX Pro-inflammatory Panel I Human Kit (Mesoscale, 4-plex, no. K15052D), according to manufacturer's guidelines.

#### 2.1.8 Adipose tissue and skeletal muscle gene expression

Microarray analysis was performed on SM and AT samples, as described previously [24]. Total RNA was extracted from adipose tissue and skeletal muscle biopsies using the Trizol method (Qiagen, Venlo, Netherlands). Subsequently, 100 ng of intact total RNA was processed applying the GeneChip® WT PLUS Reagent Kit (Affymetrix) and Human Transcriptome Array (HTA) 2.0 GeneChip (Affymetrix) according to the manufacturer's manual. Gene set enrichment analysis was based upon FDR q-value <0.20 on the filtered data set (IQR > 0.2 [log2], intensity >20, >5 arrays, >5 probes per gene), using the databases Kyoto Encyclopedia of Genes and Genomes, Reactome, Wikipathways, and Biocarta. The present data have been deposited in NCBI's Gene Expression Omnibus and are accessible through the GEO Series accession number (accession number: to be added after manuscript acceptance)

#### 2.1.9 Skeletal muscle protein expression

To evaluate the effect of MIH on (p)-Akt, (p)-AMPK, and OXPHOS complex protein expression in skeletal muscle, we performed a Western Blot analysis on protein lysates derived from the biopsies. Proteins were lysed, as described previously [25], and were subsequently homogenized. Next, a BCA assay was performed to determine protein lysate concentrations. Equal amounts of protein were loaded ([p]AMPK and [p]Akt 25 μg, OXPHOS 7.5 μg) and separated on the different gels. Primary antibodies included mouse monoclonal antibodies cocktail directed against human OXPHOS (dilution: 1:10.000; no. ab110411/no. MS601, Abcam/MitoSciences), rabbit anti-human AMPKα (1:1000, no. 2603, Cell Signaling), rabbit anti-human p-AMPKα^Thr172^ (1:1000, no. 2535, Cell Signaling), rabbit anti-mouse Akt (1:1000, no. 9272, Cell signaling), and rabbit-anti mouse p-Akt^Ser473^ (1:1000, no. 9271, Cell Signaling). Secondary antibodies were donkey anti-mouse conjugated with IRDYe800 (OXPHOS), swine anti-rabbit HRP (AMPKα, p-Akt, and Akt), and goat anti-rabbit HRP (p-AMPKα^Thr172^). Visualization and analysis were performed using a CLx Odyssey Near Infrared Imager (OXPHOS) or ChemiDoc XRS system (AMPK, Akt) and Quantity One software. Individual intensities were quantified and are expressed in arbitrary units or derived ratios.

### 2.2 Human primary cell culture experiments

#### 2.2.1 Cell culture of human primary satellite cells

Primary human satellite cells were obtained from *m. rectus abdominis* muscle of a lean, insulin-sensitive male subject. Satellite cells were cultured using a proliferation medium consisting of low glucose (1000 mg/l) Dulbecco's Modified Eagle's Medium (DMEM, no. D6046, Sigma) supplemented with 0.05 % bovine serum albumin (BSA, no. A4503, Sigma), 1 μM Dexamethasone (no. D4902, Sigma), 16 % fetal bovine serum (FBS, no. BDC-6086, Bodinco BV), 0.5 mg/ml bovine fetuin (no. 10344-034/026, Invitrogen, Life Technologies), 1x Antibiotic-Antimycotic (no. 15240-62, Gibco, Thermo Fisher Scientific), and 0.01 μg/ml recombinant human epidermal growth factor (no. PHG0311, Gibco). At 70–80 % confluence, a differentiation medium was added containing MEM-α-Glutamax (no. 32561-029, Gibco) supplemented with 2 % FBS (Bodinco BV), 0.5 mg/ml bovine fetuin (Invitrogen, Life Technologies), and 1x Antibiotic-Antimycotic (100 units∙ml^−l^ penicillin, 100 μg∙ml^−l^ of streptomycin, and 0.25 μg∙ml^−l^ Amphotericin B [no. 15240-62, Gibco]) until the myotube formation was completed. After the differentiation, myotubes were exposed to 1, 3, and 21 % O_2_ for 24 h, as described earlier [26]. Notably, more prolonged exposure to MIH was not feasible due to the relatively short lifespan of differentiated primary human myotubes. Gas mixtures were refreshed every 8 h. Subsequently, functional experiments were performed, and the cell culture medium, protein, and RNA were collected and stored at −80 °C until the analysis.

#### 2.2.2 Cell culture of human multipotent adipose-derived mesenchymal stem cells

Human multipotent abdominal subcutaneous adipose-derived mesenchymal stem cells (hMADS) were obtained from overweight/obese men with impaired glucose metabolisms and pooled and differentiated into the adipogenic lineage. Therefore, cells were seeded at a density of 2000 cells∙cm^−2^ and cultured, as described previously [27,28]. Human multipotent adipose-derived mesenchymal stem cells (hMADS) were kept in a proliferation medium containing DMEM and Ham's F-12 (DMEM-Ham's F-12) Nutrient Mixture (no. 31330-095, Gibco), 10 % FBS (Bodinco BV), and 1x Antibiotic-Antimycotic (Gibco). At ~80 % confluence, a differentiation medium was added to the cells containing DMEM-Ham's F-12, 3 % FBS (Bodinco BV), 1x Antibiotic-Antimycotic (Gibco), 33 μM D-Biotin (no. B4693, Sigma), 17 μM d-pantothenate (no. P5155, Sigma), 0.1 μM h-insulin (no. 91077C, Sigma), 1 μM dexamethasone (no. D4902, Sigma), 250 μM 3-isobutyl-1-methylxanthine (IBMX, no. I5879, Sigma), and 5 μM rosiglitazone (no. ALX-350-125-M025, Enzo Life Sciences). After 7 days, IBMX and rosiglitazone were removed from the medium. All cells were proliferated and differentiated under 21 % O_2_, and thereafter, exposed to either 10 % O_2_ continuously (resembling physiological normoxia), or to MIH consisting of 3 × 2 h cycles per day, alternating between 5 and 10 % O_2_, during the final 7 days of cell culture.

#### 2.2.3 Glucose uptake in primary human myotubes and adipocytes

Basal and insulin-stimulated glucose uptake were determined in both primary human myotubes and differentiated hMADS following serum-starvation for 2 h, as described previously [28]. Briefly, hMADS and myotubes were serum-starved 2 h prior to the glucose uptake experiment. After two washes in modified Krebs Ringer buffer (1.17 M NaCl, 26 mM KCl, 12 mM KH_2_PO_4_, 12 mM MgSO_4_, 100 mM NaHCO_3_, 100 mM HEPES, 0.1 % BSA, and 1 mM CaCl_2_), cells were incubated for 30 min in 20 μM 2-Deoxy-d-glucose and 55 μM (0.44 μCi/ml [^3^H-]-2-Deoxy-d-glucose [#NET328A001MC, Perkin Elmer]) at 37 °C. Subsequently, h-insulin was added, and the cells were incubated for another 30 min. Cells were then scraped and lysed in 0.05 M NaOH. Subsequently, 200 μL of the cell lysate was transferred into glass scintillation vials containing 5 ml OptiFluor (Perkin Elmer). β-decay was measured using a liquid scintillation counter (Perkin Elmer), reflecting the cellular uptake of (^3^H-)-2-Deoxy-d-glucose.

To investigate the involvement of adenosine monophosphate-activated kinase (AMPK) in the effects of hypoxia on glucose uptake in human primary myotubes, we co-incubated cells with and without 10 μM Compound C (no. P5499, Sigma) during hypoxia exposure and 1 mM AICAR (no. A9978, Sigma) as the positive control. Next, basal glucose uptake was measured.

#### 2.2.4 Myotube protein expression

After exposure to 1, 3, and 21 % O_2_ for 24 h, protein lysates were collected. Subsequently, protein concentrations were identified, and p-AMPKαThr^172^, AMPKα, and OXPHOS complex protein expression were determined by Western Blotting. Briefly, 1x RIPA buffer supplemented with a protease/phosphatase inhibitor cocktail (Cell Signaling Technology Europe, Leiden, The Netherlands) was added to lyse the myotubes. After scraping the cells, the protein lysate was collected, and the protein concentration was determined (BCA Protein Assay kit; Santa-Cruz Biotechnology Inc.). 15 μg protein was separated on BioRad Criterion TGX gels (Bio-Rad Laboratories; 12 % for OXPHOS, Any-kD for other targets) and subsequently blotted using Trans-Blot Turbo Transfer System (Bio-Rad; for AMPKα, OXPHOS, and GAPDH) and Bio-Rad Criterion Blotter (Bio-Rad; for p-AMPKαThr^172^, GAPDH), respectively. Membranes were incubated overnight at 4 °C with primary antibodies in corresponding blocking buffers according to the manufacturer's protocols. Subsequently, blots were incubated with secondary antibodies for 1 h at room temperature. The primary antibodies used were AMPKα (no. 2603, Cell Signaling), p-AMPKαThr^172^ (no. 2535, Cell Signaling), total OXPHOS antibody cocktail (no. MS601, Mitosciences), and GAPDH (no. 2118, Cell Signaling). Secondary antibodies used were swine-anti-rabbit HRP (no. P0339, DAKO), rabbit-anti-mouse HRP (no. P0161, DAKO), and goat-anti-rabbit HRP (no. PI-1000, Vector Laboratories). Antigen-antibody complexes were visualized by chemiluminescence using SuperSignal™ West Femto and Dura extended Duration Substrates (Life Technologies). Visualization and analysis were performed using a ChemiDoc XRS system (Bio-Rad) and Quantity One software.

#### 2.2.5 Adipokine secretion

At the end of each exposure regimen, the differentiated adipocytes and myotubes medium was collected to determine adipokine medium concentrations of IL-6, MCP-1, leptin, adiponectin, and VEGF using ELISA, as described previously [27].

#### 2.2.6 Adipocyte and myotube gene expression

To obtain RNA for gene expression analysis, we added TRIzol Reagent (Invitrogen) to the cells at the end of exposure, and RT-PCR was performed, as described previously [27]. Firstly, the extracted RNA was precipitated and purified according to the manufacturer's protocol. Subsequently, cDNA was synthesized out of purified RNA using RT-PCR (iScript cDNA synthesis kit, no. 170–8891, Bio-Rad). Next, SYBR-Green-based real-time PCRs were performed using an iCycler (Bio-Rad; primer sequences are provided in Supplementary Table 3). Results were normalized to the geometric mean of 18S ribosomal RNA and RPL13A.

### 2.3 Statistics

The sample size was calculated based on a physiologically relevant 20 % change of peripheral insulin sensitivity (α = 0.05, 1-β = 0.9). Data were checked for normality by the Shapiro–Wilk test. The effects of MIH (compared to normoxia exposure) were assessed by a paired Student's *t*-test, whereas nonparametric data were analyzed using the Wilcoxon Signed-Rank test. Areas under the curves were calculated using the trapezoidal method. *In vitro* experiments were analyzed using Wilcoxon Signed-Rank tests or the Friedman's test with a *post-hoc* Dunn's test. Data are expressed as means ± standard error of the mean (SEM), with a two-sided significance level of *p* < 0.05. The statistical analysis was performed using SPSS 24.0 for Macintosh. Figures were created using Graphpad Prism.

## 3 Results

### 3.1 Subject characteristics

Twelve overweight and obese men with homeostasis model assessment for insulin resistance (HOMA_IR_) ≥2.2 and without any chronic disease or endocrine disorder participated in the present randomized, single-blind crossover study. Subjects were recruited between April 2016 and December 2019. One participant did not undergo the two-step hyperinsulinemic-euglycemic clamp procedure. Participants’ characteristics are shown in Table 1.

**Table 1:** Baseline characteristics of study participants.

	Baseline
Age (y)	61 ± 1
BMI (kg/m^2^)	30.8 ± 0.9
Hemoglobin (mmol∙l^−1^)	9.5 ± 0.5
HbA_1c_ (mmol∙mol^−1^)	37.9 ± 0.9
HbA_1c_ (%)	5.6 ± 0.1
Fasting glucose (mmol∙l^−1^)	5.7 ± 0.2
2h-glucose (mmol∙l^−1^)	6.2 ± 0.4
HOMA_IR_	3.7 ± 0.4

BMI, body mass index; HbA_1c_, glycated hemoglobin; HOMA_IR_, Homeostatic Model of Assessment of Insulin Resistance. Values are represented as mean ± SEM (*n* = 12).

### 3.2 Mild intermittent hypoxia exposure reduces systemic oxygen saturation and partial oxygen pressure in adipose tissue and skeletal muscle

**Figure 1:**
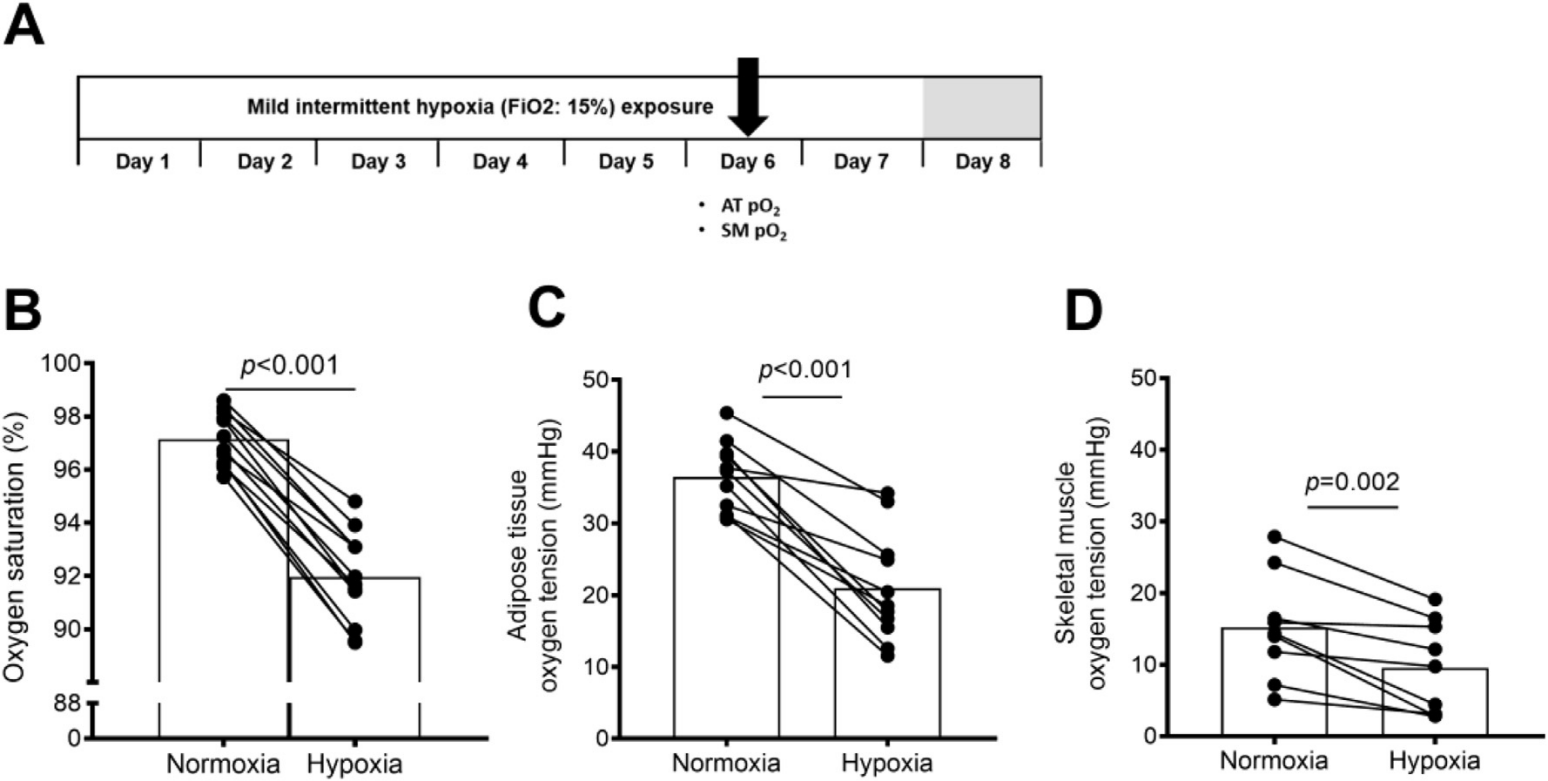
**Mild intermittent hypoxia exposure decreases systemic oxygen saturation, adipose tissue, and skeletal muscle oxygen tension.** Adipose tissue and skeletal muscle oxygen tension pO_2_ were determined during MIH exposure at day 6 (**A**). MIH exposure significantly decreased (**B**) systemic oxygen saturation, (**C**) adipose tissue, and (**D**) skeletal muscle pO_2_. Data are represented as mean (bars) and individual data points. Statistical analysis was performed using a two-tailed Student's paired t-test.

MIH exposure significantly reduced SpO_2_ (normoxia: 97.1 ± 0.3 *vs*. hypoxia: 92.0 ± 0.5%, *p* < 0.001), and markedly decreased AT pO_2_ (normoxia: 36.5 ± 1.5 mmHg versus hypoxia: 21.0 ± 2.3 mmHg, *p* < 0.001) and SM pO_2_ (normoxia: 15.4 ± 2.4 mmHg versus hypoxia: 9.5 ± 2.2 mmHg*; p* = 0.002; Figure 1).

These findings provide the first proof-of-concept in humans that MIH exposure not only reduces systemic oxygen saturation but also consistently decreases AT and SM pO_2_ by ~40 %. In addition, SM pO_2_ was significantly lower than AT pO_2_ (*p* < 0.001), likely reflecting the higher metabolic rate in SM. No adverse events were reported.

### 3.3 Mild intermittent hypoxia exposure induces a shift toward glycolytic metabolism

**Figure 2:**
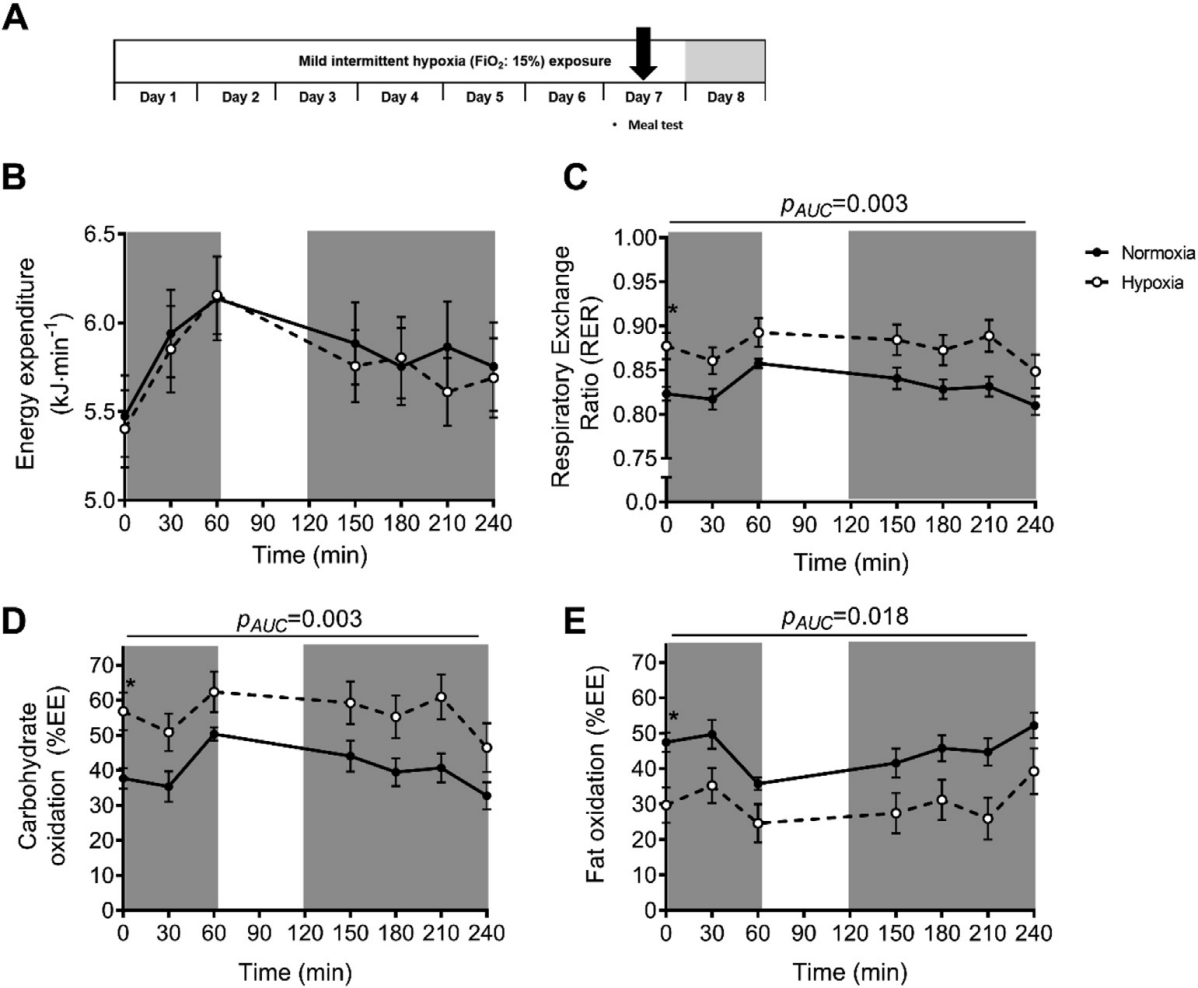
**Mild intermittent hypoxia exposure alters fasting and postprandial substrate oxidation.** Indirect calorimetry was performed before (t = 0 min) and after ingestion of a high-fat mixed meal (t = 0–240 min) to determine energy expenditure substrate oxidation during mild intermittent hypoxia (MIH) exposure (**A**). MIH exposure did not alter (**B**) energy expenditure but significantly increased fasting and postprandial (**C**) respiratory exchange ratio and (**D**) carbohydrate oxidation (% of total energy expenditure: EE%) and decreased (**E**) fat oxidation compared to normoxia exposure. Total area under the curves (AUC) but not incremental AUCs (iAUC) were significantly different between conditions. Open circles/dashed line, MIH exposure; closed circles/solid line, normoxia exposure. Gray areas indicate time periods when study participants resided in the hypoxic room. Data are represented as mean ± SEM. Statistical analysis was performed using a two-tailed Student's paired t-test.

Fasting and postprandial energy expenditure (Figure 2B) were not significantly altered by MIH, though fasting energy expenditure tended to be reduced (*p* = 0.053). MIH induced a pronounced increase in the fasting respiratory exchange ratio (RER, *p* = 0.001) and carbohydrate oxidation (CHO, *p* = 0.002), whereas fat oxidation was reduced (FAO, *p* = 0.013) compared to normoxia exposure. Moreover, following meal intake, RER remained elevated (*p*_AUC_ = 0.003, Figure 2C) during MIH compared to normoxia exposure due to a marked shift toward increased postprandial CHO (*p*_AUC_ = 0.003, Figure 2D) and suppression of FAO (*p*_AUC_ = 0.018, Figure 2E).

**Figure 3:**
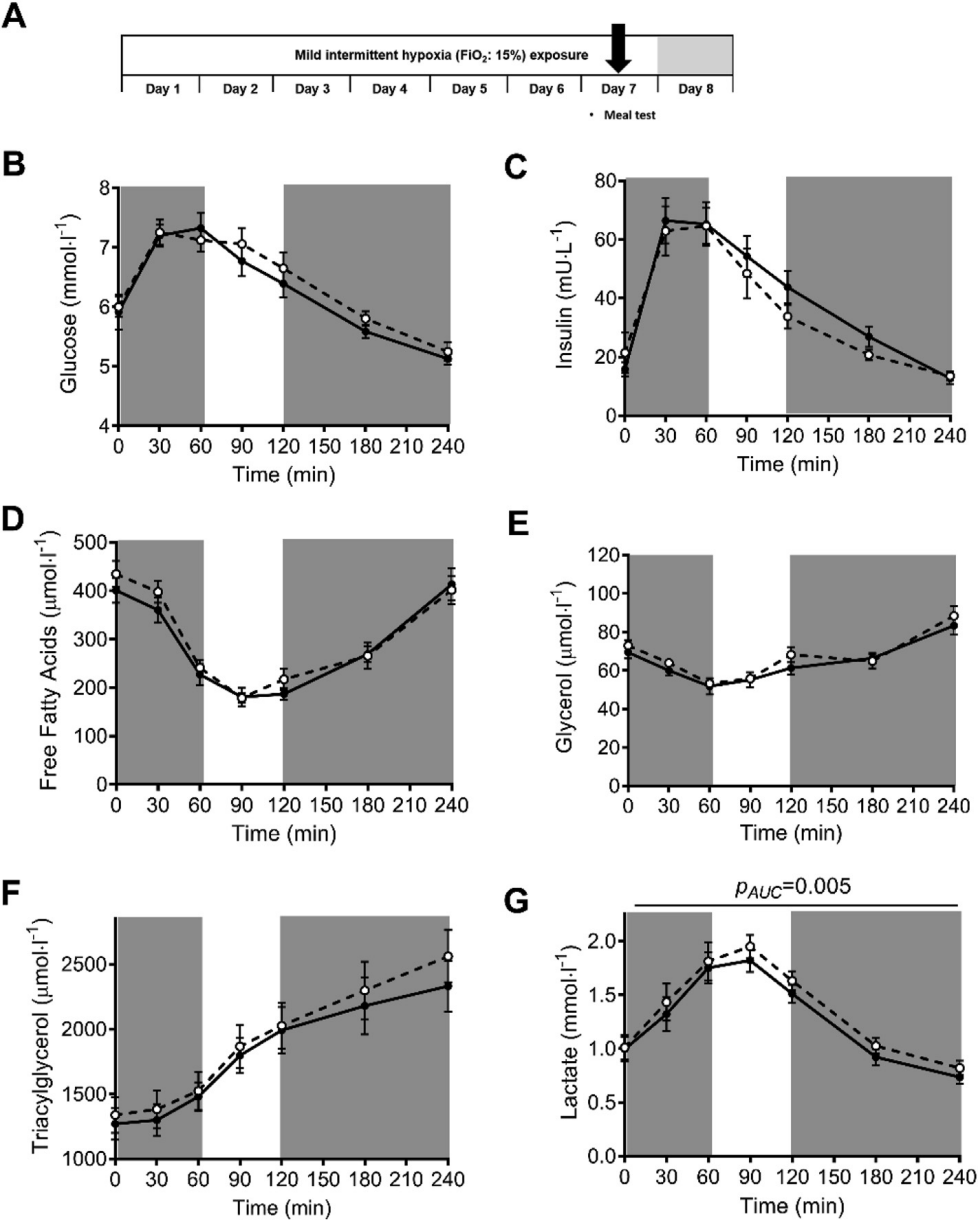
**The effects of mild intermittent hypoxia exposure on fasting and postprandial plasma metabolites.** Mild intermittent hypoxia exposure during the high-fat mixed meal challenge **(A)** did not significantly alter fasting (t = 0 min) and postprandial (t = 0–240 min) plasma concentrations of **(B)** glucose, **(C)** insulin, **(D)** free fatty acids, **(E)** glycerol, and **(F)** triacylglycerol but significantly increased **(G)** postprandial plasma lactate concentrations. Total areas under the curves (AUC) but not incremental AUCs (iAUC) were significantly different between conditions. Open circles/dashed line, MIH exposure; Closed circles/solid line, normoxia exposure. Grey areas indicate time periods when study participants resided in the hypoxic room. Data are represented as mean ± SEM. Statistical analysis was performed using the nonparametric Wilcoxon's signed-rank test.

In addition, MIH exposure did not alter fasting and postprandial plasma glucose, insulin, free fatty acid, triacylglycerol, or glycerol concentrations compared to normoxia exposure (Figure 3B–F). Nevertheless, plasma lactate levels were increased by MIH compared to normoxia (*p*_AUC_ = 0.005, Figure 3G). Notably, the MIH-induced effects on fasting substrate utilization and plasma lactate concentrations were maintained but not more pronounced during postprandial conditions, as MIH did not alter the incremental areas under the curves (iAUC∙min^−1^) for these parameters (Figure 3).

### 3.4 Mild hypoxia exposure increases basal glucose uptake in human primary myotubes via AMPK

**Figure 4:**
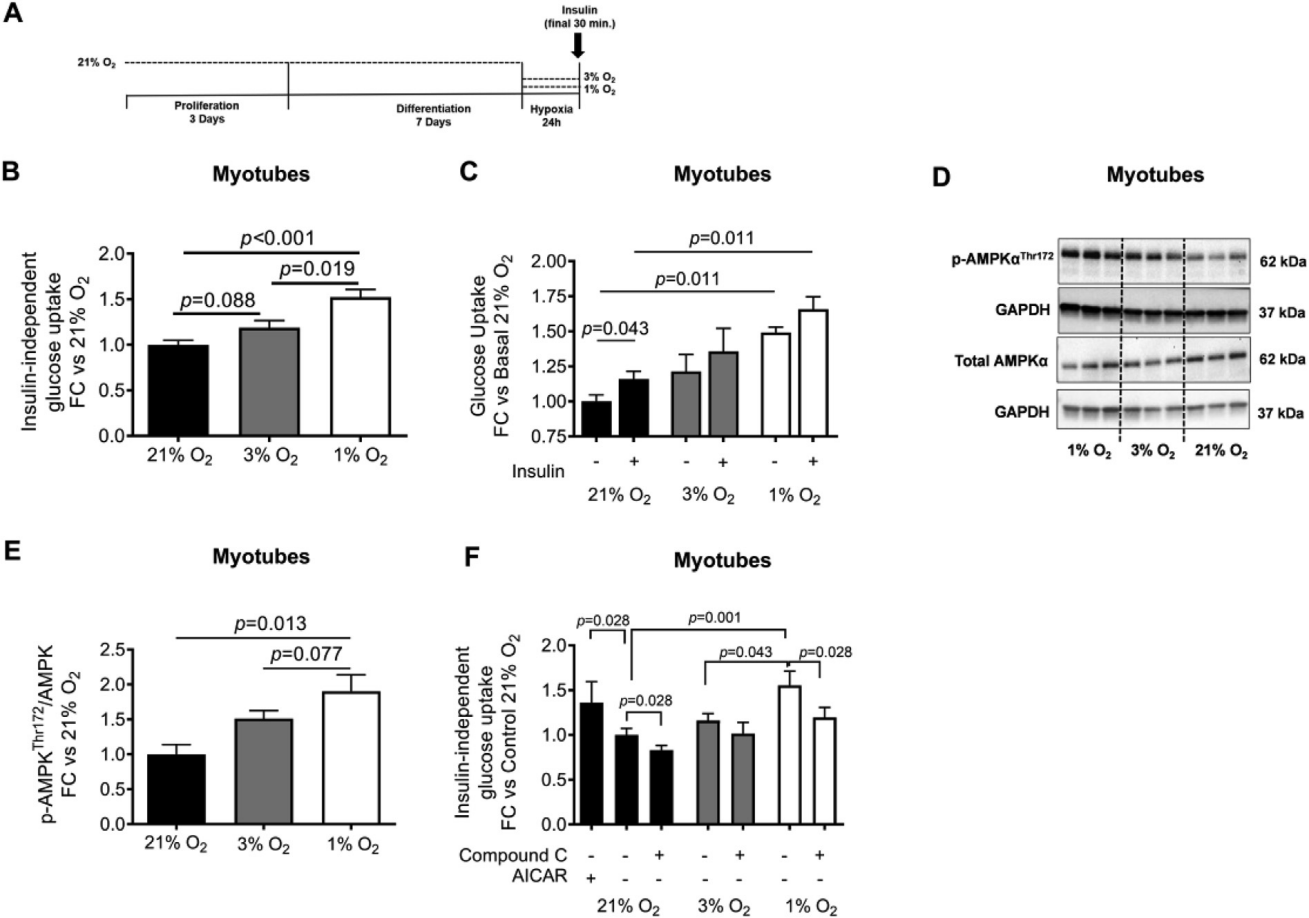
**Hypoxia exposure increases insulin-independent glucose uptake in primary human myotubes.** Primary human myotubes were cultured at oxygen tension reflecting physiological hypoxia (1 % O_2_) and normoxia (3 % O_2_) in human skeletal muscle, as well as standard lab conditions (21 % O_2_), for 24 h to investigate the effects on glucose uptake and involvement of AMPK herein (**A**). Exposure to 1 % O_2_ increased (**B**) insulin-independent (basal) glucose uptake but did not alter (**C**) the increase in glucose uptake under insulin-stimulated conditions (100 nM insulin) in primary human myotubes compared to 3 % and 21 % O_2_. (**D, E**) Hypoxia exposure increased p-AMPK^Thr172^/AMPK protein expression compared to standard lab conditions and (**F**) co-incubation experiments using the AMPK inhibitor Compound C (dorsomorphin, 10 μM) and the AMPK agonist AICAR (1 mM; positive control under 21 % O_2_) demonstrated that the hypoxia-induced increase in insulin-independent glucose uptake is mediated through AMPK activation in human primary myotubes. Black bars, 21 % O_2_; gray bars, 3 % O_2_; white bars, 1 % O_2_ exposure. Data are represented as mean ± SEM, **(B)***n* = 11, **(C)***n* = 5, **(D, E)***n* = 4, **(F)***n* = 6. Statistical analysis was performed using nonparametric Wilcoxon's signed-rank test and Friedman's test using Dunn's *post-hoc* multiple comparison test. Compound C, dorsomorphin; AICAR, 5-Aminoimidazole-4-carboxamide ribonucleotide; FC, fold change; GAPDH, Glyceraldehyde 3-phosphate dehydrogenase.

Human primary myotubes were exposed to oxygen levels that we found *in vivo* during MIH and normoxia exposure (Figure 4A and Figure 1C). As expected, mRNA expression levels of the hypoxia-responsive genes *VEGFA* (*p* = 0.007 and *p* = 0.003), *GLUT1* (*p* = 0.047 and *p* = 0.013), *BNIP3* (*p* = 0.003 and *p* = 0.006), and *CA-9* (*p* = 0.056 and *p* = 0.056) were markedly increased following hypoxia exposure (1 % O_2_) compared to both 3 % and 21 % O_2_, respectively (Supplementary Figure 2). Furthermore, 1 % O_2_ markedly increased glucose uptake compared to 3 % O_2_ (*p* = 0.019, Figure 4B) and 21 % O_2_ (*p* < 0.001, Figure 4B). Similar findings were observed after 30 min of insulin stimulation, with significantly increased insulin-mediated glucose uptake after 1 % O_2_ compared to 21 % O_2_ (*p* = 0.011, Figure 4C). Nevertheless, the insulin-induced increase in glucose uptake (insulin-stimulated – basal) did not significantly differ between exposure conditions (Figure 4C).

In addition, exposure to 1 % O_2_ increased the p-AMPK^Thr172^/AMPK ratio in human myotubes compared to 3 % O_2_ (*p* = 0.077) and 21 % O_2_ (*p* = 0.013; Figure 4D,E). The inhibition of AMPK by co-incubation with Compound C during 1 % O_2_ exposure decreased hypoxia-induced glucose uptake (*p* = 0.028) to levels found after 3 % O_2_ exposure (Figure 4F).

### 3.5 Mild intermittent hypoxia exposure does not affect tissue-specific insulin sensitivity but induces a metabolic shift toward increased carbohydrate oxidation

**Figure 5:**
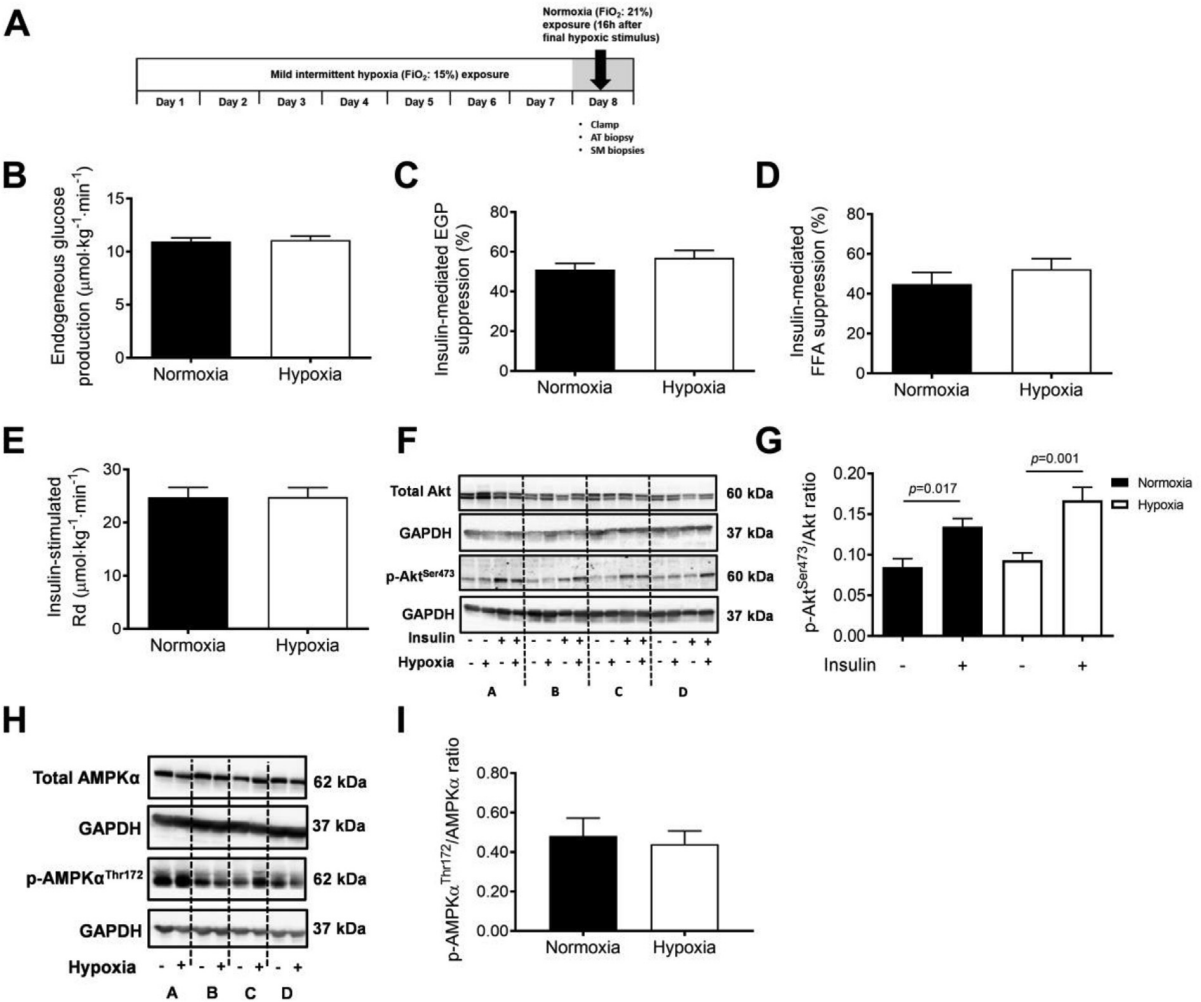
**Mild intermittent hypoxia exposure does not alter hepatic, adipose tissue, and peripheral insulin sensitivity.** Tissue-specific insulin sensitivity was determined by a two-step hyperinsulinemic-euglycemic clamp under normoxic conditions after 7 days of MIH exposure (**A**). MIH does not significantly affect (**B**) fasting endogenous glucose production (EGP) by the liver, (**C**) the suppression of EGP (%), (**D**) and the suppression of plasma free fatty acid concentration (%) during the steady state of low-insulin infusion (10 mU∙m^−2^∙min^−1^), and (**E**) insulin-stimulated rate of glucose disposal during the steady state of high-insulin infusion (40 mU∙m^−2^∙min^−1^). (**F**) p-Akt^Ser473^ and Akt protein expression and (**G**) p-Akt^Ser473^/Akt ratio in fasting and insulin-stimulated (40 mU∙m^−2^∙min^−1^) skeletal muscle biopsies (representative western blot of 4 participants indicated (participants A, B, C, and D). **(H)** Fasting p-AMPKα^Thr172^ and AMPKα protein expression, and **(I)** ratio in fasting skeletal muscle biopsies. Data are represented as mean (bars) ± SEM. Statistical analysis was performed using a two-tailed Student's paired t-test.

The two-step hyperinsulinemic-euglycemic clamp with a D-[6,6-^2^H_2_]-glucose tracer infusion (Figure 5A) revealed that MIH did not significantly alter basal endogenous glucose production (Figure 5B), hepatic insulin sensitivity (suppression of endogenous glucose production [EGP] during low-insulin infusion, 10 mU∙m^−2^∙min^−1^; Figure 5C), or AT insulin sensitivity (insulin-mediated suppression of free fatty acids during low-insulin infusion, 10 mU∙m^−2^∙min^−1^; Figure 5D) compared to normoxia. Moreover, peripheral insulin sensitivity (insulin-stimulated glucose rate of disappearance, Rd) was not significantly affected by MIH (Figure 5E). In addition, non-oxidative glucose disposal (NOGD) remained unaltered following MIH compared to normoxia exposure.

In agreement with these findings, we found that MIH did not induce changes in SM insulin signaling, determined using fasting (baseline) and insulin-stimulated (40 mU∙m^−2^∙min^−1^) SM biopsies collected before and during the hyperinsulinemic-euglycemic clamp, respectively. More specifically, the insulin-induced increase in the p-Akt^Ser473^/total Akt protein expression ratio in skeletal muscle was comparable following MIH and normoxia exposure (Figure 5F,G). In addition, MIH did not change the muscle p-AMPK^Thr172^/AMPK ratio compared to normoxia (*p* = 0.346; Figure 5H,I).

Intriguingly, the fasting RER (*p* = 0.050) and CHO (*p* = 0.050) remained increased at day 8, whereas FAO remained reduced (*p* = 0.049), indicating that MIH impacts substrate utilization for at least 16 h after the cessation of the last bout of hypoxia (Supplementary Figure 3). In line with these findings, at day 8 (normoxia), the RER (*p* = 0.070) and CHO (*p* = 0.095) continued to be increased, whereas FAO tended to be reduced (*p* = 0.091) during high-insulin infusion. However, insulin-induced changes in substrate oxidation rates were not altered, suggesting that metabolic flexibility was not affected by MIH.

### 3.6 Mild intermittent hypoxia exposure does not alter skeletal muscle oxidative capacity

**Figure 6:**
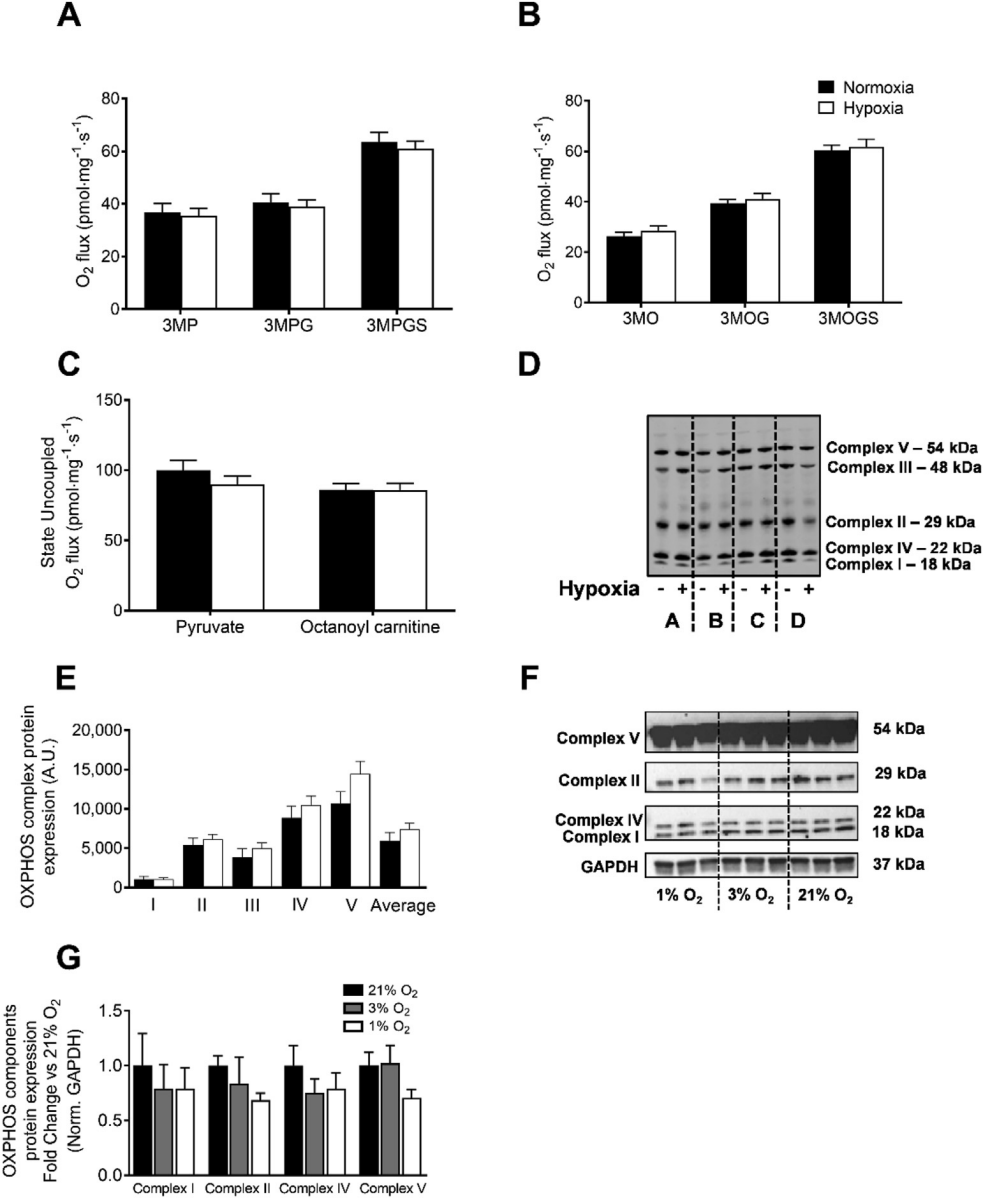
**Mild intermittent hypoxia exposure does not affect skeletal muscle oxidative capacity.** A skeletal muscle biopsy was obtained from the *m. vastus lateralis* under fasting conditions. *Ex vivo* mitochondrial respiration experiments were performed to determine (**A**) State 3 ADP-stimulated respiration upon a carbohydrate-like (pyruvate), (**B**) lipid-like (octanoyl-carnitine) substrate with parallel electron input into complex I and II, and (**C**) maximal uncoupled respiration upon stimulation with FCCP in both leads. (**D**) OXPHOS components complex (I–V) protein expression in skeletal muscle biopsies collected under fasting conditions (representative western blot of 4 participants, indicated by A-D). (**E**) Quantification of OXPHOS protein expression. (**F**) Primary human myotubes were cultured at oxygen tension reflecting physiological hypoxia (1 % O_2_) and normoxia (3 % O_2_) in human skeletal muscle, as well as standard lab conditions (21 % O_2_), for 24 h to investigate the effects of hypoxia exposure on OXPHOS protein expression. (**G**) Quantification of OXPHOS protein expression in myotubes. Closed circles, normoxia exposure; open circles, MIH exposure. Data are represented as mean ± SEM. 3, state 3; M, malate; G, glutamate; S, succinate; P, pyruvate; O, octanoyl-carnitine; FCCP, Carbonyl cyanide-4-(trifluoromethoxy)phenylhydrazone. I–V; Complex I–V proteins; A.U., arbitrary units.

MIH did not induce alterations in state 3 and maximally uncoupled mitochondrial respiration using carbohydrate- and lipid-leads (Figure 6A–C). In agreement with these findings, the protein expression of OXPHOS complex components was unchanged following MIH (Figure 6D,E). In line with these findings, western blot analysis revealed that mild hypoxia exposure did not affect OXPHOS complex protein expression in human primary myotubes (Figure 6F,G).

### 3.7 Mild intermittent hypoxia exposure impacts inflammatory, metabolic, and ECM-related pathways in adipose tissue but not skeletal muscle

**Figure 7:**
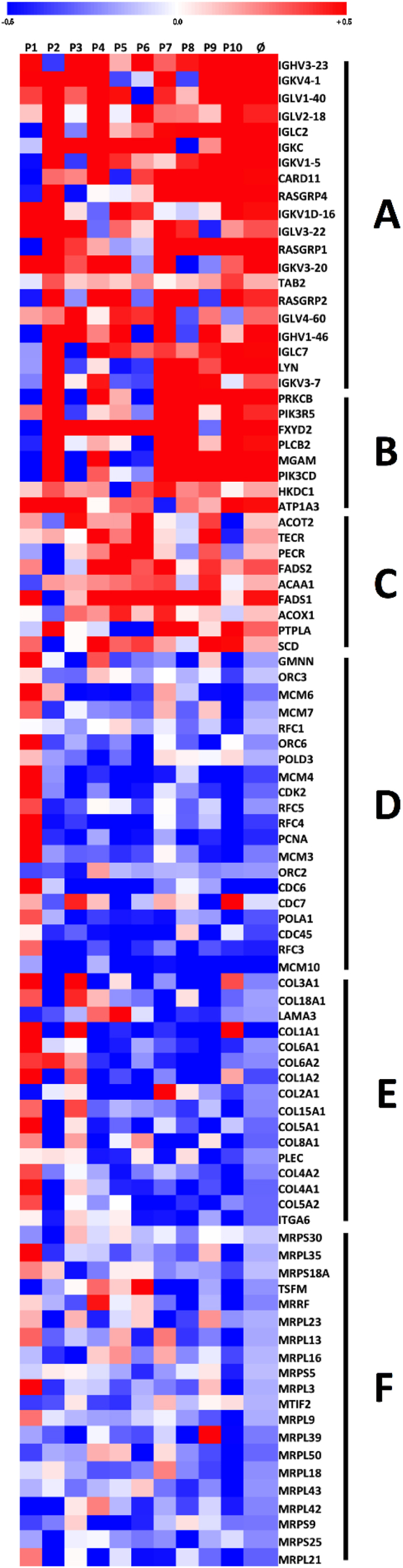
**Mild intermittent hypoxia exposure alters gene expression in human adipose tissue.** Adipose tissue biopsies were collected on day 8 to determine the effects of MIH exposure compared to normoxia exposure for seven consecutive days on adipose tissue expression. Gene set enrichment analyses were performed (signal log ratios: hypoxia – normoxia), and signal to log ratios are depicted per subject and as overall mean (Ø). Genes most significantly contributing to up- and down-regulated pathways following MIH compared to normoxia exposure were selected (false discovery rate, *q*-value <0.2, *p* < 0.05). Colors reflect the individual signal to log ratios, with red being upregulated and blue being downregulated compared to normoxia exposure. (**A**) FCERI-mediated NF-κB activation. (**B**) Carbohydrate digestion and absorption. (**C**) Biosynthesis of unsaturated fatty acids. (**D**) WP466: DNA replication. (**E**) WP2798: Assembly of collagen fibrils and other multimeric structures. (**F**) Mitochondrial translation. A list of all pathways significantly altered by MIH exposure is shown in Supplementary Table 1.

In total, 171 gene sets were positively (and 285 negatively) enriched in AT following 7-day MIH compared to normoxia. In contrast, no significantly differentially expressed pathways could be identified in SM after gene set enrichment analysis (GSEA) at FDR *q*-value <0.2, *p* < 0.05 (all affected pathways in AT and individual genes in SM are in Supplementary Tables 1 and 2, respectively). In AT, pathways related to inflammation and carbohydrate and lipid metabolism were significantly upregulated, reflected by positive normalized enrichment scores. The expression of gene sets related to the cell cycle, mitochondrial translation, and extracellular matrix components was downregulated, reflected by negative normalized enrichment scores (Figure 7).

Interestingly, we found that MIH downregulated the collagen assembly pathway, which may reflect reduced ECM stress, possibly reflective of the initiation of AT remodeling. Furthermore, we found that MIH upregulated pathways related to inflammation transcription factor nuclear factor-κB (NF-κB) and various downstream cytokine signaling pathways involving cytokines, such as interleukin-1 (IL-1) and IL-2, and complement factors. However, systemic levels of inflammatory cytokines tumor necrosis factor α (TNFα), interferon-γ (IFN-γ), IL-6, and IL-8 were not affected by MIH exposure (Supplementary Figure 4).

### 3.8 Mild hypoxia exposure affects adipokine expression and secretion but not glucose uptake in primary human adipocytes

MIH exposure reduced VEGF (*p* = 0.005) and leptin (*p* = 0.034) secretion by primary human adipocytes compared to 10 % and 21 % O_2_, respectively, but did not significantly affect IL-6, MCP-1, or adiponectin secretion (Supplementary Figure 5A–E). In line with reduced leptin secretion, leptin mRNA expression was significantly reduced by MIH exposure compared to 21 % O_2_ (*p* = 0.005; Supplementary Figure 5F). In contrast, MIH increased VEGFA mRNA expression compared to 10 % O_2_ (*p* = 0.011).

Moreover, we determined the impact of MIH exposure on glucose uptake in primary human adipocytes. MIH did not significantly alter insulin-(in)dependent glucose uptake compared to 10 % O_2_ (Supplementary Figure 5G).

### 3.9 Mild intermittent hypoxia induces slight hemodynamic adaptations but does not alter feelings of hunger and satiety

MIH exposure did not affect SBP and DBP, despite a slight but significant increase in mean heart rate (*p* = 0.017, Supplementary Figure 6). Finally, we found that feelings of hunger, satiety, and thirst were not significantly affected by MIH exposure, determined by Visual Analogue Scales (VAS) completed by the participants before and after breakfast, lunch, and dinner (Supplementary Figure 7).

## 4 Discussion

In the present randomized, controlled, single-blind crossover study, we demonstrated for the first time that MIH exposure for 7 consecutive days reduces systemic oxygen saturation, decreases AT and SM pO_2_, evokes a shift toward glycolytic metabolism, and induces adaptations in AT and SM but does not impact AT, hepatic, or SM insulin sensitivity in men with obesity.

We found that MIH exposure markedly increased whole-body carbohydrate oxidation, an effect that persisted after the cessation of the exposure regimen until the following day at the least. This shift in substrate oxidation was also reflected by increased plasma lactate concentrations during the high-fat mixed-meal test under MIH, indicating an increased glycolytic rate. Importantly, MIH exposure did not induce hyperventilation, suggesting that the increased RER found reflects increased carbohydrate oxidation and does not seem to be explained by respiratory alkalosis. In accordance with our findings, it has been previously demonstrated that short-term mild (hypobaric) hypoxia exposure increased reliance on carbohydrate oxidation [[29], [30], [31]] but did not affect postprandial glucose and insulin levels [32].

Mechanistically, the present data demonstrate that hypoxia exposure increased glucose uptake via an AMPK-dependent mechanism in primary human myotubes but not adipocytes, in line with previous *in vitro* studies [15,[33], [34], [35]]. In contrast to our *in vitro* findings in primary human myotubes and previous observations in rodent skeletal muscle [36,37], we did not find altered p-AMPK^Thr172^/AMPK protein expression in human SM following MIH exposure. As the muscle biopsies were collected ~16 h after the last hypoxic stimulus, we cannot exclude that AMPK was already dephosphorylated at the time of sample collection.

Interestingly, the present randomized, controlled, single-blind crossover study demonstrates that for 7 days, MIH did not alter AT, hepatic, or SM insulin sensitivity. In contrast, findings from previous studies in rodents [[11], [12], [13], [14]] and humans [15] have shown that prolonged exposure to mild hypoxia improves insulin sensitivity and/or glucose homeostasis. However, it is important to emphasize that the latter finding was based on a within-group comparison, as a control group was not included in the study design [15].

Moreover, our data show that MIH increased the AT gene expression of inflammatory/metabolic pathways and decreased extracellular matrix-related pathways. However, MIH effects on adipokine expression/secretion did not translate into changes in low-grade systemic inflammation. Conflicting findings have been reported on the effects of (severe) hypoxia on inflammatory processes, which seems to be related to the severity, pattern, and duration of hypoxia exposure [2]. We have recently shown that prolonged exposure to mild hypoxia reduces the expression of pro-inflammatory genes in cultured human adipocytes [27]. An alternative explanation for the upregulation of inflammation-related pathways in AT in the present study may include hypoxia-induced lactate production, contributing to increased NF-κB-related gene expression, as observed in L6 myocytes and macrophages [38,39]. Conversely, the hypoxia–lactate axis might mitigate inflammation by suppressing macrophage activation and polarization toward anti-inflammatory M2-macrophages [40,41].

Furthermore, *ex vivo* SM mitochondrial respiratory capacity and the OXPHOS protein expression in SM were unaltered following MIH exposure. The hypoxia-induced activation of hypoxia-inducible factor (HIF)-1α activation might inhibit decarboxylation of pyruvate into acetyl-CoA by pyruvate dehydrogenase [42]. This effect may then shunt pyruvate away from the mitochondria, resulting in reduced mitochondrial respiration and enhancing glycolysis to ensure sufficient ATP production [42]. Notably, in the present study, the SM biopsies used to isolate the SM fibers used to determine oxygen consumption rates were collected ~16 h after the last hypoxic stimulus of the exposure regimen and, therefore, reflect the chronic rather than the acute response of MIH on the oxidative machinery in human SM.

The strengths of the present study are that this study is the first randomized crossover trial investigating the effects of MIH exposure on many cardiometabolic risk factors in humans to our knowledge. Importantly, measurements were performed under well-controlled conditions, and participants were kept in energy balance throughout the study. Furthermore, we studied the isolated effects of MIH under normobaric conditions, thereby excluding the possible effects of a different ambient pressure present at high altitudes (i.e., hypobaric hypoxia). Finally, we performed extensive (functional) measurements in tissue biopsies and human primary myotubes and adipocytes, cultured under oxygen levels that mimic the adipose tissue and skeletal muscle microenvironments in humans to explore underlying mechanisms.

Noteworthy, several nuances have to be made regarding the conclusions of the present study. First, as we studied men with obesity characterized by modest impairments in glucose homeostasis, we cannot exclude that MIH exposure may have other effects in individuals with a different metabolic phenotype (i.e., normal-weight individuals, patients with type 2 diabetes) or women. Secondly, the duration of the exposure to MIH was relatively short (42 h in total) due to the invasive nature of the study and the burden on the participants. Therefore, we cannot exclude that more prolonged exposure to mild (intermittent) hypoxia or exposure to more severe hypoxia is required to induce beneficial effects on glucose homeostasis, and this possibility warrants further investigation. Thirdly, as the primary human myotubes were continuously exposed to different oxygen levels for 24 h, we cannot exclude that exposure to an MIH regimen mimicking the human *in vivo* study (i.e., 3 × 2 h per day) would have yielded less pronounced increases in AMPK activation and glucose uptake.

## 5 Conclusions

In summary, the findings of the present randomized, single-blind crossover trial demonstrate that 7 consecutive days of MIH exposure decreases AT and SM pO_2_, induces a metabolic switch toward increased reliance on glycolysis to ensure ATP production and evokes adaptations in AT and SM in humans with obesity. However, these findings do not translate into alterations in AT, hepatic, or SM insulin sensitivity. Further studies are warranted to investigate whether other hypoxia exposure regimens (longer intervention period and/or more severe hypoxic episodes) and/or pharmacological strategies targeting oxygen signaling may mitigate obesity-related metabolic perturbations. For example, the stabilization of HIF by prolyl-hydroxylase (PHD) inhibitors improved glucose tolerance, serum cholesterol, and inflammation in rodents [43,44]. Noteworthy, PHD inhibitors have recently been used in patients with chronic kidney disease [45], but the putative effects of these agents on metabolic homeostasis have not been examined yet to our knowledge.

## Author contributions

G.G. supervised this work. R.V.M., M.V., E.B., and G.G conducted hypothesis generation, conceptual design, and data analysis. R.V.M., M.V., L.V., J.J, J.S. N.H., J.H., Y.E., P.S., H.S., S.K., and G.G. contributed to data acquisition. G.G. acquired funding for the work. R.V.M., M.V., and G.G. wrote the manuscript, and G.G. had the primary responsibility for the final content. G.G is the guarantor of this work and, as such, had full access to all the data in the study and takes responsibility for the integrity of the data and the accuracy of the data analysis. All authors revised the content of the manuscript and read and approved the final version of the manuscript for publication.

## Funding

This study was supported by a Senior Fellowship grant from the 10.13039/501100003092Dutch Diabetes Research Foundation (grant number: 2015.82.1818) and a Rising Star Award Fellowship (2014) from the 10.13039/501100001648European Foundation for the Study of Diabetes to G.G.

## Data and materials availability

The datasets generated by microarray analysis are deposited at the Gene Expression Omnibus (GEO) database (accession number < to be added upon acceptance>). Furthermore, primer sequences used in this study are included in Supplementary Table 3.
