# Supplementary material for: Mild intermittent hypoxia exposure induces metabolic and molecular adaptations in men with obesity
Source: Mol Metab. 2021 Jul 3;53:101287. doi: 10.1016/j.molmet.2021.101287 (PMC8355948; doi:10.1016/j.molmet.2021.101287)
Supplement: Multimedia component 1 [file mmc1.docx]

**Supplementary Material**

**Mild Intermittent Hypoxia Exposure Induces Metabolic and Molecular Adaptations in Obese Men**

Rens L.J. van Meijel^1,^‡, Max A.A. Vogel^1,^‡, Johan W.E. Jocken^1^, Lars M.M. Vliex^1^, Joey S.J. Smeets^1^, Nicole Hoebers^1^, Joris Hoeks^2^, Yvonne Essers^1^, Paul F.M. Schoffelen^1, 2^, Henrike Sell^3^†, Sander Kersten^4^, Kasper M.A. Rouschop^5^, Ellen E. Blaak^1^, Gijs H. Goossens^1^*

**Supplementary Figures**

**
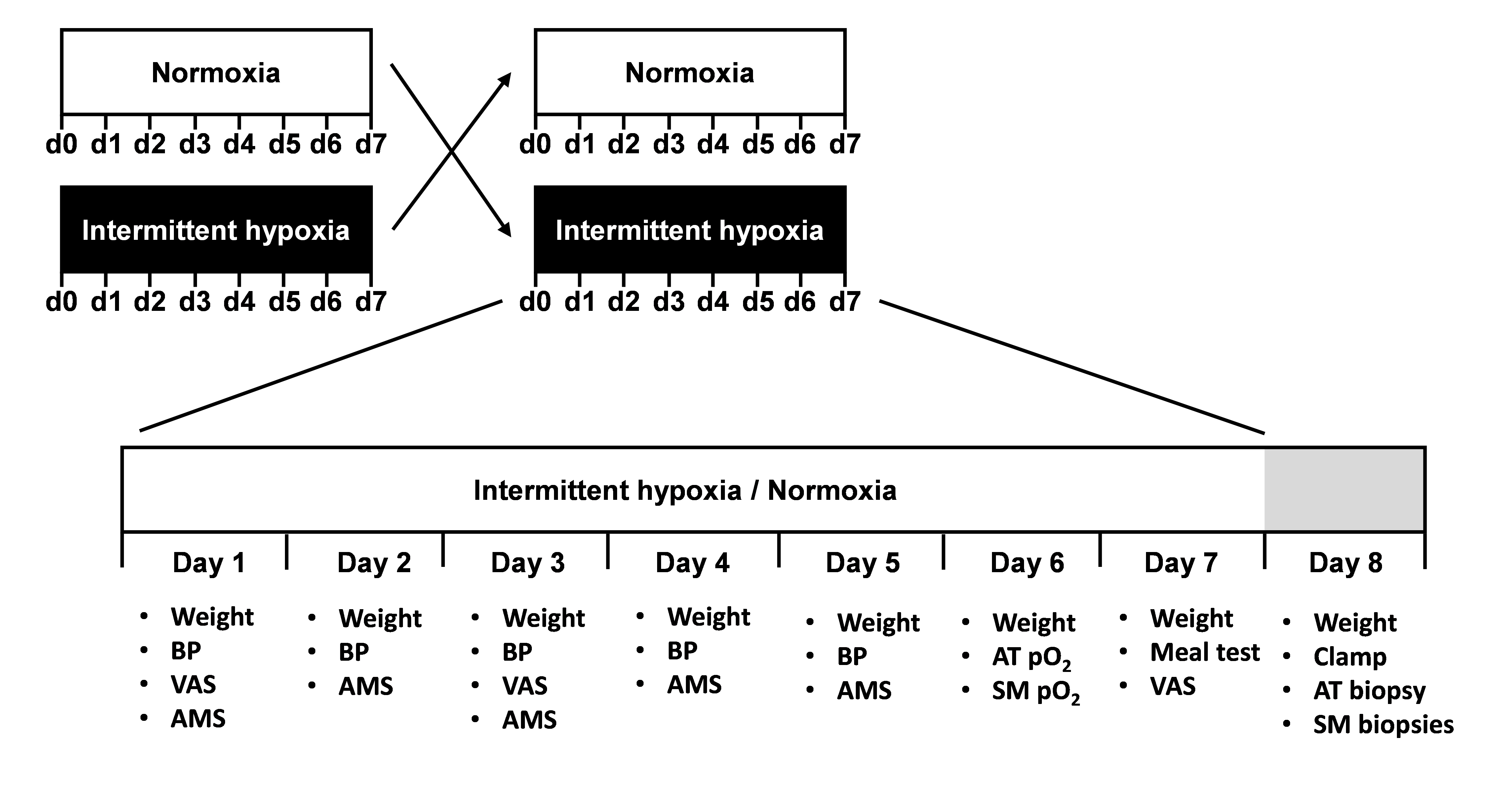
 Supplementary Figure 1. Study flowchart of the randomized, single-blind crossover trial.** Participants were exposed to normoxia (21% O_2_) and mild intermittent hypoxia (15% O_2_, 3x2h per day) with a 3-6 week washout period, in a randomized fashion. At day 8, a two-step hyperinsulinemic-euglycemic clamp was performed under normoxic conditions, during both exposure regimens. BP, blood pressure; VAS, visual analog scale questionnaire; AMS, acute mountain sickness questionnaire; AT pO_2_, adipose tissue; SM, skeletal muscle; pO_2_, oxygen tension.


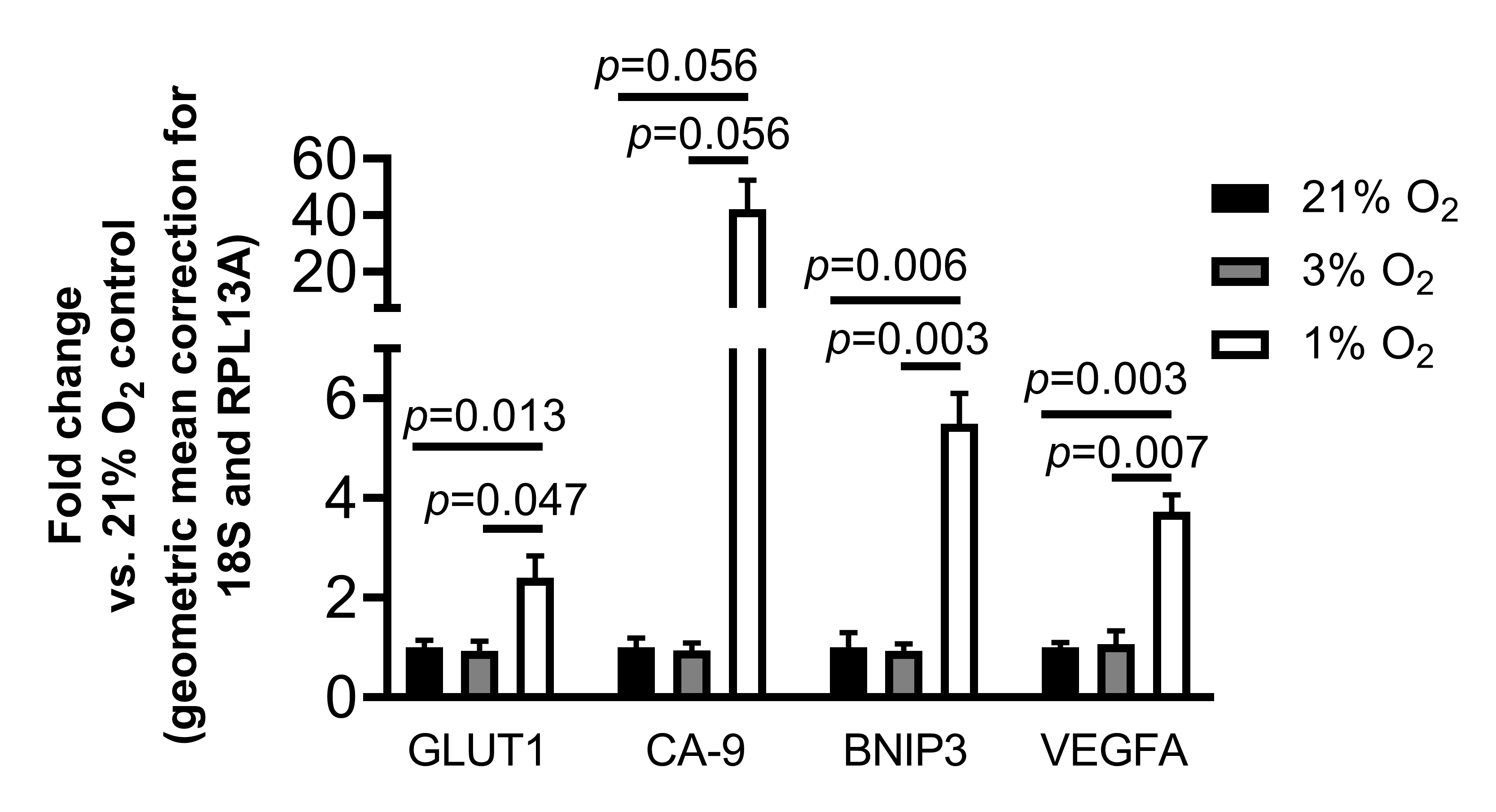


**Supplementary Figure 2. Hypoxia exposure increased expression of several hypoxia-responsive genes in primary human myotubes.** Gene expression (mRNA) of *GLUT1*, *CA-9*, *BNIP3* and *VEGFA* was quantified and expressed as fold change compared to 21% O_2_, and corrected for the geometric mean of *18S* and *RPL13A* expression. *GLUT1*, glucose transporter 1; *CA-9*, carbonic anhydrase 9; *BNIP3*, BCL2 Interacting Protein 3; *VEGFA*, vascular endothelial growth factor-a. 21% O_2_, *n*=4; 3% O_2_, *n*=4; 1% O_2_, *n*=3. Statistical analysis was performed using two-tailed Student’s paired t-test. The exact *p*-values are shown.


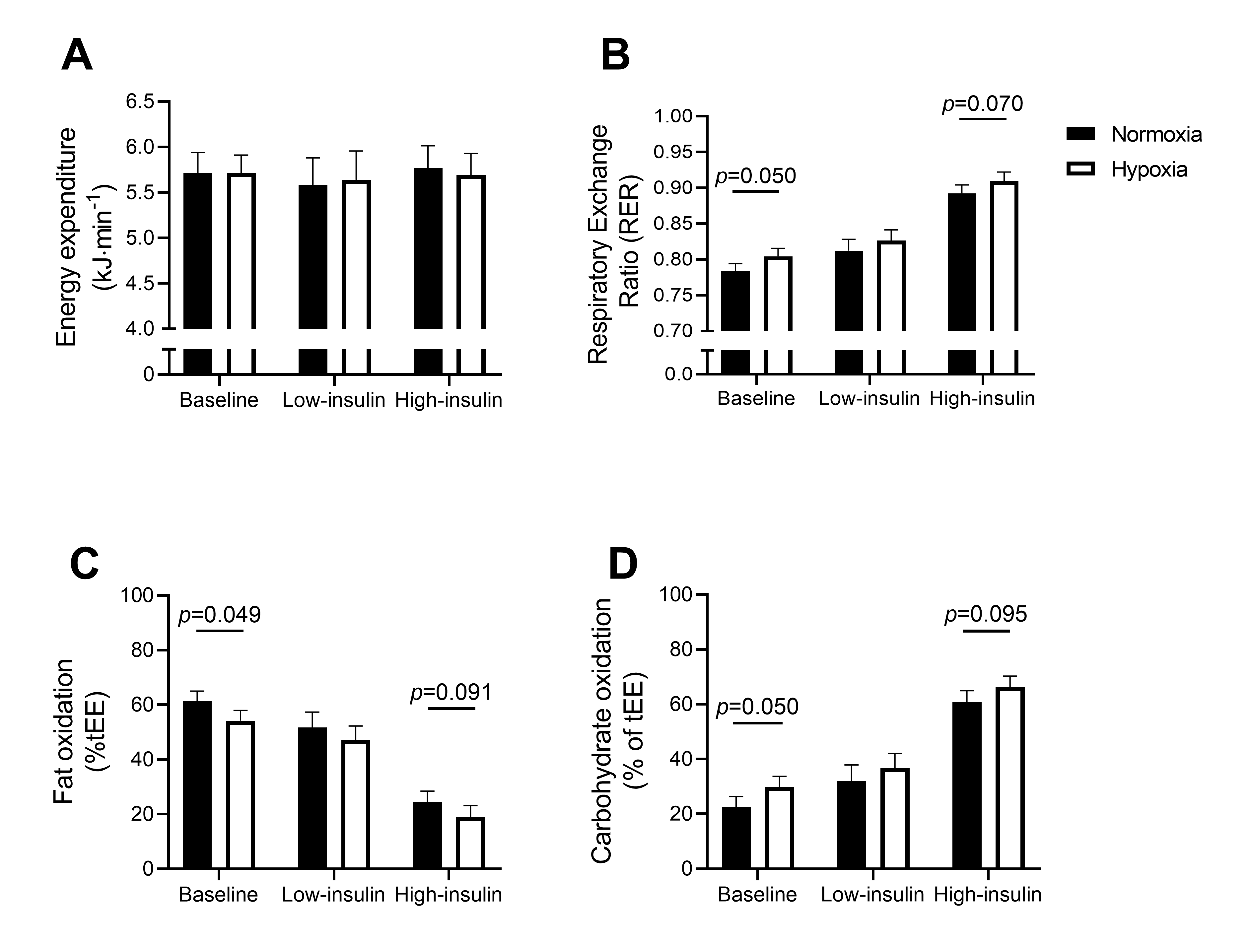


**Supplementary Figure 3. Substrate oxidation during the hyperinsulinemic-euglycemic clamp (day 8).** Fasting and insulin-stimulated (low-insulin: 10 mU∙m^-2^∙min^-1^; high-insulin: 40 mU∙m^-2^∙min^-1^) energy expenditure and substrate oxidation was measured by indirect calorimetry during the steady state phases of a two-step hyperinsulinemic-euglycemic clamp. MIH exposure did not alter energy expenditure **(A)** but increased fasting respiratory exchange ratio **(B)**, which was reflected by reduced fat oxidation **(C)** and increased carbohydrate oxidation **(D)** compared to normoxia exposure. Closed circles, normoxia exposure; open circles, MIH exposure. Values are represented as individual data (connected lines). Statistical analysis was performed using two-tailed Student’s paired t-test. The exact *p*-values are shown. %EE, percentage of total energy expenditure.


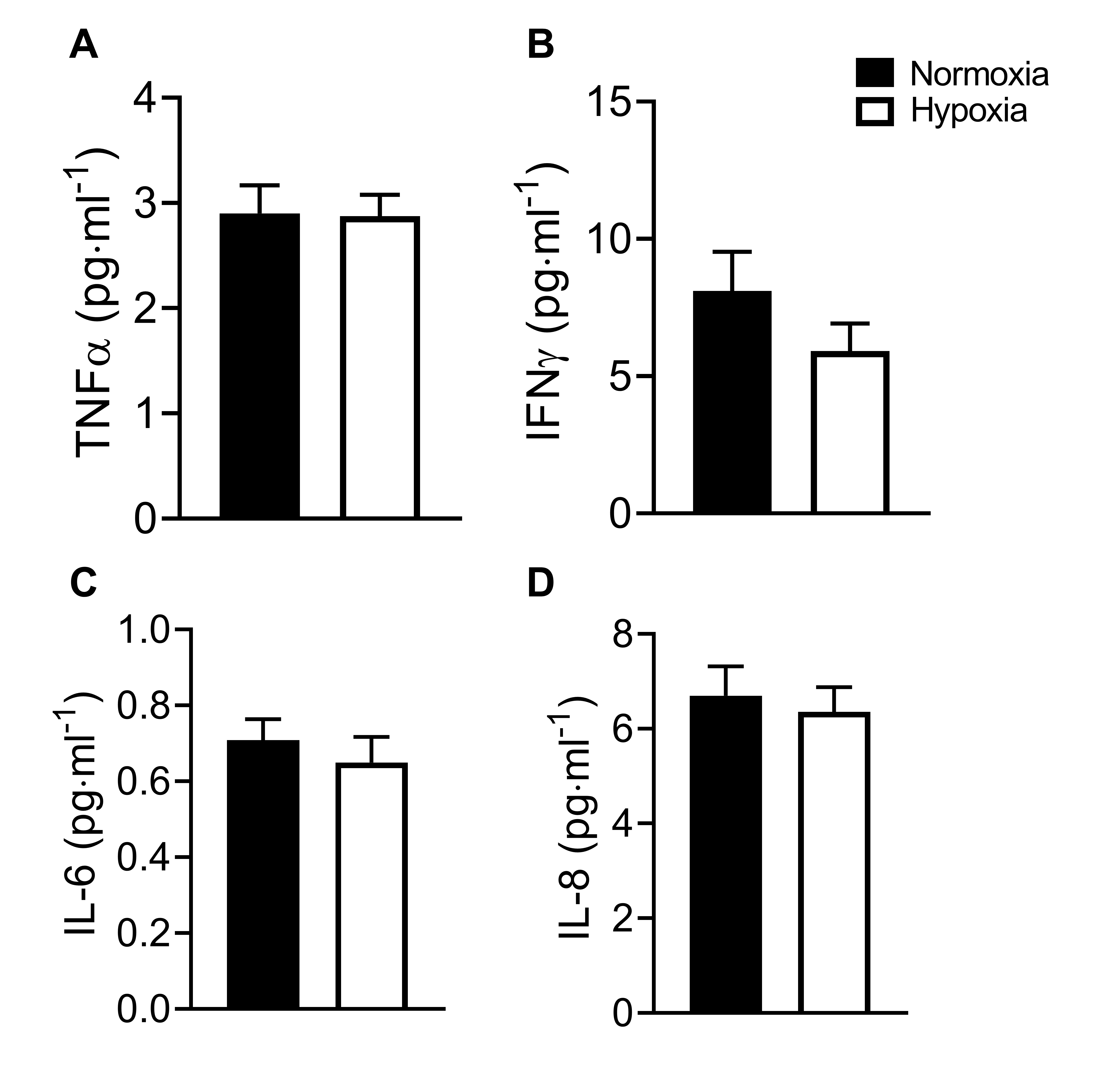

**Supplementary Figure 4. The effect of mild intermittent hypoxia compared to normoxia exposure on systemic concentrations of inflammatory cytokines.** Mild intermittent hypoxia (MIH) exposure did not significantly alter fasting plasma concentrations of **(A)** TNFα, **(B)** IFN-γ, **(C)** IL-6 and **(D)** IL-8, determined at day 8, subsequent to MIH exposure. Black and white bars represent normoxic and MIH conditions, respectively. Data are represented as mean ± SEM. TNFα, tumor necrosis factor α; IFN-γ, interferon-γ; IL, interleukin.

**
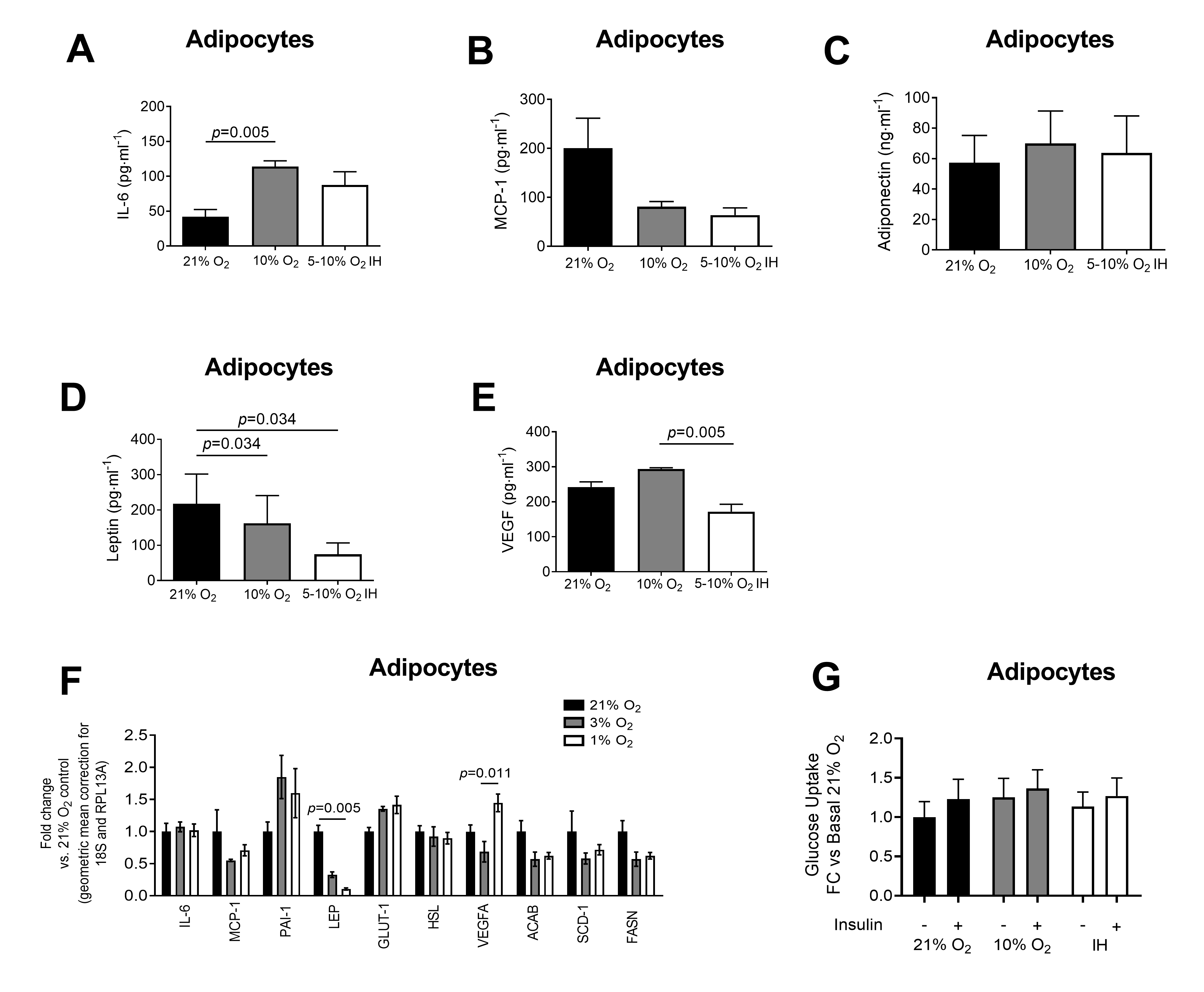
**

**Supplementary Figure 5. The effect of mild intermittent hypoxia compared to normoxia exposure on adipokine secretion and gene expression in primary human adipocytes.** Adipose tissue-derived mesynchymal stem cells were differentiated for 14 days. During days 7-14, the cells were exposed to mild intermittent hypoxia exposure (from 10% to 5% O_2_, 3x2h per day) or continuous exposure to 10% O_2_ and 21% O_2_, resembling normoxia and standard lab conditions, respectively. Secretion of (**A**) IL-6, (**B**) MCP-1, (**C**) adiponectin, (**D**) leptin and (**E**) VEGF were measured after 7 days of exposure to the different oxygen levels. (**F**) Gene expression (mRNA) of *IL-6*, *DPP-4*, *MCP-1*, *PAI-1*, *LEP*, *GLUT-1*, HSL, *VEGFA*, *ACAB*, *SCD1* and *FASN* was quantified and expressed as fold change compared to 21% O_2_, and corrected for the geometric mean of 18S and RPL13A expression. **(G)** Differentiated primary human adipocytes exposed to MIH for 7 days (alternating cycles of 10-5% O_2_, 3x2h per day) did not alter basal and insulin-stimulated glucose uptake compared to a fixed oxygen level at 10% O_2_ and 21% O_2_, resembling normoxia in human adipose tissue and standard laboratory conditions, respectively. Black bars, 21% O2; grey bars 10% O2; white bars, 5-10% O2, intermittent hypoxia. Data are represented as mean ± SEM, **(A-E)** *n* = 4, **(F)** *n* = 4-5, **(G)** *n* = 4. Statistical analysis was performed using Friedman’s test with Dunn’s *post hoc* multiple comparison test. The exact *p*-values are shown. *IL-6*, Interleukin-6; *MCP-1*, monocyte chemoattractant protein-1; *PAI-1*, plasminogen activator inhibitor-1; *LEP*, leptin; *GLUT1*, glucose transporter 1; *HSL*, hormone sensitive lipase; *VEGF*, vascular endothelial growth factor; *ACAB*, acetyl-CoA acetyltransferase; *SCD-1*, stearoyl-CoA desaturase-1; *FASN*, fatty acid synthase; *18S*, 18S ribosomal RNA; *RPL13A*, ribosomal protein L13a.


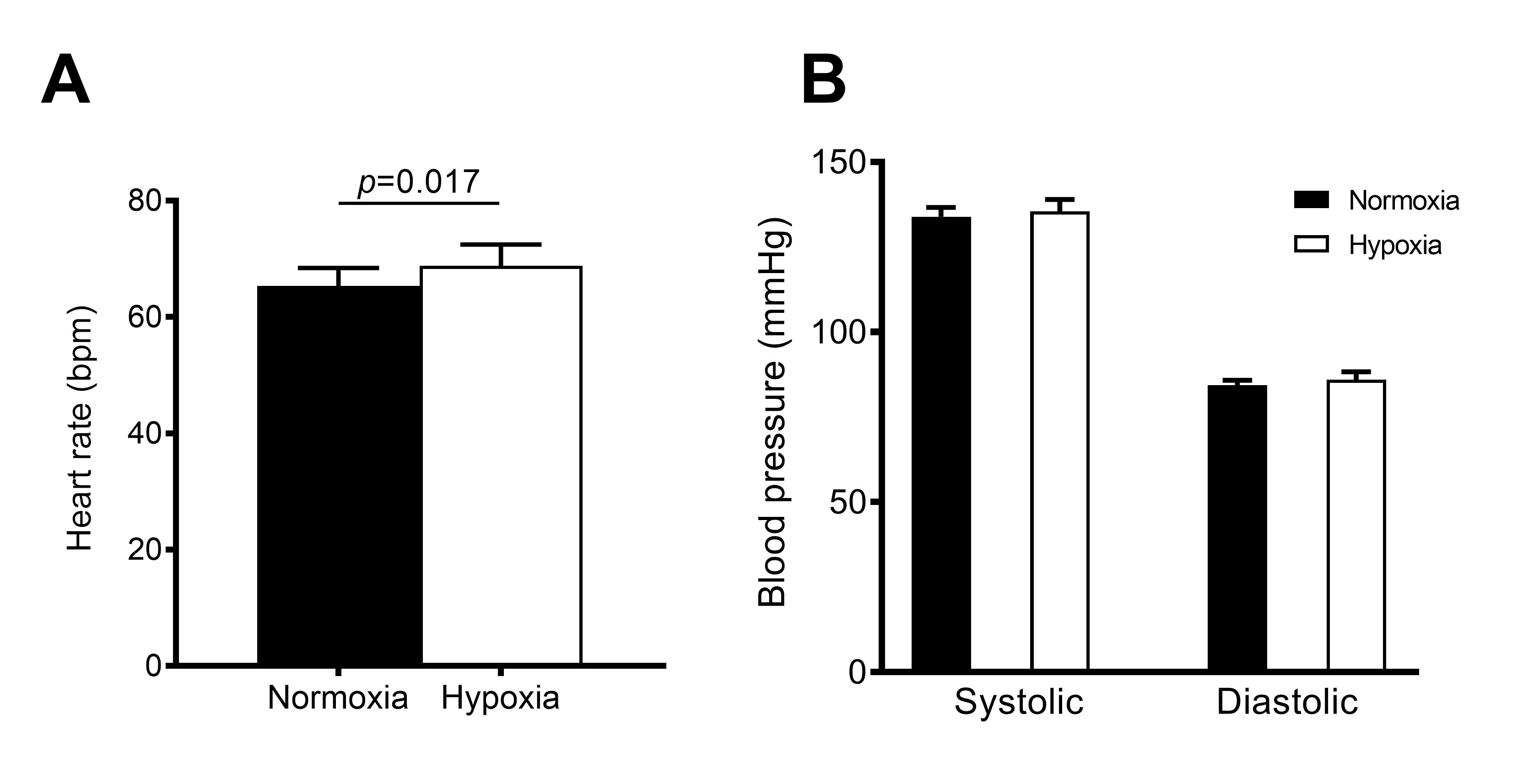


**Supplementary Figure 6.** **The effects of mild intermittent hypoxia compared to normoxia exposure on heart rate, systolic and diastolic blood pressure.** Heart rate and blood pressure (average values on days 1-5) were measured under fasting conditions. (**A**) MIH exposure significantly increased HR but (**B**) did not induce changes in systolic and diastolic blood pressure. Closed circles, normoxia exposure; open circles, MIH exposure. Data are represented as mean ± SEM. Statistical analysis was performed using two-tailed Student’s paired t-test. The exact *p*-values are shown.

**
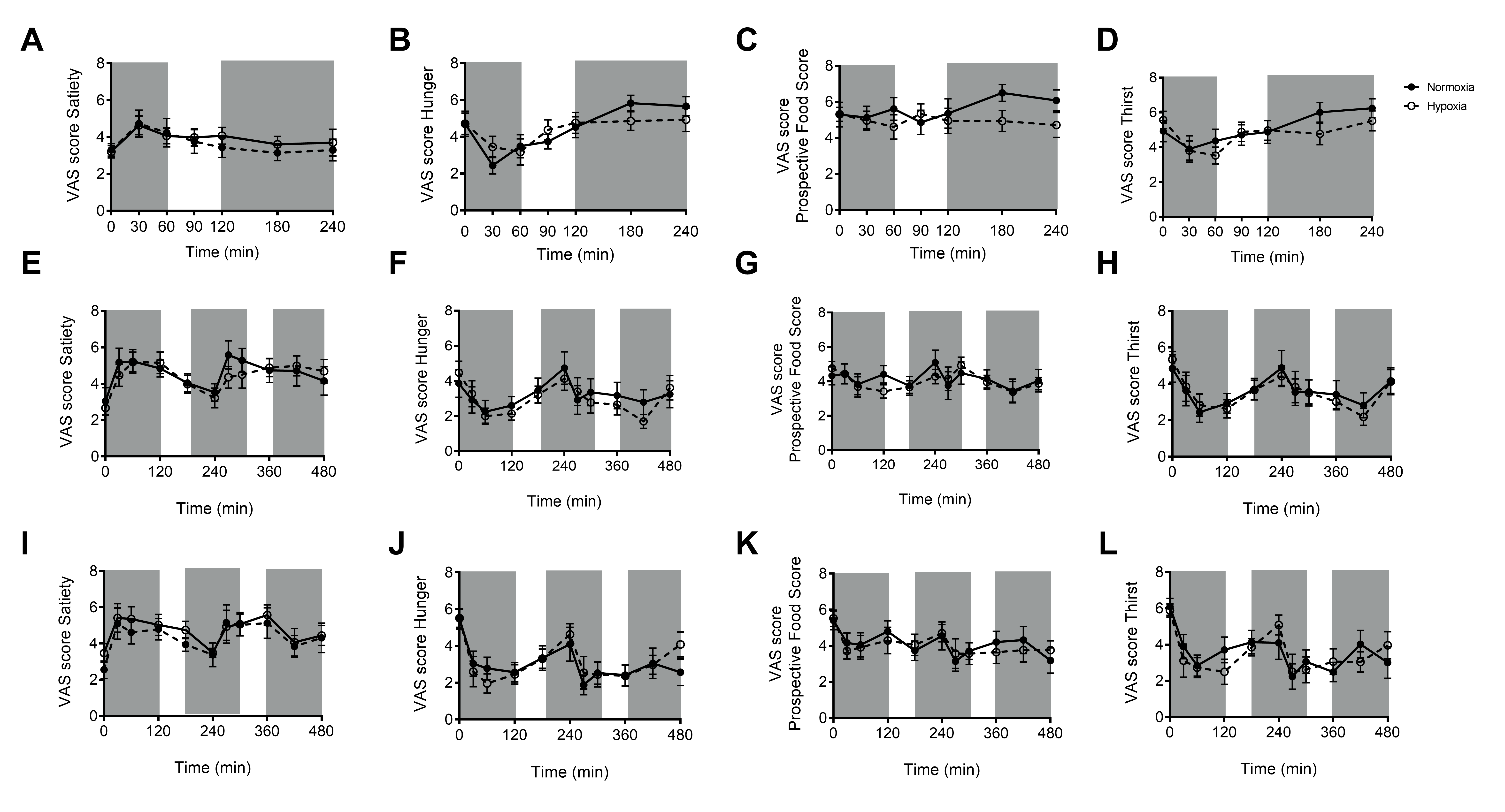
**

**Supplementary Figure 7.** **The effects of mild intermittent hypoxia exposure on thirst, hunger and satiety feelings.** At days 1, 3, and 7, visual analog scale questionnaires were completed by the study participants to assess feelings of thirst, hunger and satiety, and prospective food consumption scores. (**A-D**) Feelings of thirst, hunger and satiety determined during the high-fat mixed meal challenge (day 7: meal intake at t=0 min) . Feelings of thirst, hunger and satiety determined before and after breakfast (t=0 min), lunch (t=240 min) and dinner (t=480 min) on day 1 (**E-H**) and day 3 (**I-L**). Satiety scores (**A**, **E,** **I**), hunger scores (**B**, **F**, **J**), prospective food consumption scores (**C, G, K**), and thirst scores (**D, H, L**) were not significantly different between MIH and normoxia exposure. Open circles / dashed line, MIH exposure; Closed circles / solid line, normoxia exposure. Grey areas indicate time periods when study participants were inside the hypoxic room. Data are represented as mean ± SEM.

**Supplementary Tables**

### **Supplementary Table 1**. List of significantly upregulated (positive values) and downregulated (negative values) pathways by mild intermittent hypoxia exposure in abdominal subcutaneous adipose tissue identified with gene set enrichment analyses (n=10).

| **Gene set** | | | **NES** | **FDR q-value** | |  |  |  |  |  |
| --- | --- | --- | --- | --- | --- | --- | --- | --- | --- | --- |
|  |  |  |  |  |  |  |  |  |  |  |
| **Mild intermittent hypoxia exposure - upregulated** | | |  |  | |  |  |  |  |  |
| FCGR.ACTIVATION | | | 2,52 | 0 | |  |  |  |  |  |
| IMMUNOREGULATORY.INTERACTIONS.BETWEEN.A.LYMPHOID.AND. A.NON.LYMPHOID.CELL | | | 2,49 | 0 | |  |  |  |  |  |
| FCERI.MEDIATED.NF.KB.ACTIVATION | | | 2,48 | 0 | |  |  |  |  |  |
| ROLE.OF.PHOSPHOLIPIDS.IN.PHAGOCYTOSIS | | | 2,42 | 0 | |  |  |  |  |  |
| SCAVENGING.OF.HEME.FROM.PLASMA | | | 2,34 | 0 | |  |  |  |  |  |
| KEGG_NATURAL.KILLER.CELL.MEDIATED.CYTOTOXICITY | | | 2,3 | 0 | |  |  |  |  |  |
| WP1829.IMMUNOREGULATORY.INTERACTIONS.BETWEEN.A.LYMPHOID. AND.A.NON.LYMPHOID.CELL | | | 2,3 | 0 | |  |  |  |  |  |
| CREATION.OF.C4.AND.C2.ACTIVATORS | | | 2,25 | 0 | |  |  |  |  |  |
| KEGG_B.CELL.RECEPTOR.SIGNALING.PATHWAY | | | 2,24 | 0 | |  |  |  |  |  |
| ANTIGEN.ACTIVATES.B.CELL.RECEPTOR.BCR.LEADING.TO. GENERATION.OF.SECOND.MESSENGERS | | | 2,23 | 0 | |  |  |  |  |  |
| CLASSICAL.ANTIBODY.MEDIATED.COMPLEMENT.ACTIVATION | | | 2,22 | 0 | |  |  |  |  |  |
| WP23.B.CELL.RECEPTOR.SIGNALING.PATHWAY | | | 2,17 | 0 | |  |  |  |  |  |
| WP1927.TCR.SIGNALING | | | 2,16 | 0 | |  |  |  |  |  |
| FCERI.MEDIATED.CA.2.MOBILIZATION | | | 2,16 | 0 | |  |  |  |  |  |
| INITIAL.TRIGGERING.OF.COMPLEMENT | | | 2,15 | 0 | |  |  |  |  |  |
| TCR.SIGNALING | | | 2,14 | 0 | |  |  |  |  |  |
| WP69.TCR.SIGNALING.PATHWAY | | | 2,08 | 0 | |  |  |  |  |  |
| FCERI.MEDIATED.MAPK.ACTIVATION | | | 2,07 | 0 | |  |  |  |  |  |
| KEGG_OSTEOCLAST.DIFFERENTIATION | | | 2,05 | 0 | |  |  |  |  |  |
| BIOC_NKCELLSPATHWAY | 1,99 | | | 0,01 | |  |  |  |  |  |
| FCGAMMA.RECEPTOR.FCGR.DEPENDENT.PHAGOCYTOSIS | 1,98 | | | 0,01 | |  |  |  |  |  |
| WP2732.INTERLEUKIN.2.SIGNALING | 1,98 | | | 0,01 | |  |  |  |  |  |
| WP2694.DAP12.INTERACTIONS | 1,97 | | | 0,01 | |  |  |  |  |  |
| REGULATION.OF.ACTIN.DYNAMICS.FOR.PHAGOCYTIC.CUP. FORMATION | 1,96 | | | 0,01 | |  |  |  |  |  |
| KEGG_LEISHMANIASIS | 1,93 | | | 0,02 | |  |  |  |  |  |
| WP2291.DEREGULATION.OF.RAB.AND.RAB.EFFECTOR.GENES. IN.BLADDER.CANCER | 1,92 | | | 0,02 | |  |  |  |  |  |
| KEGG_T.CELL.RECEPTOR.SIGNALING.PATHWAY | 1,91 | | | 0,02 | |  |  |  |  |  |
| KEGG_PRIMARY.IMMUNODEFICIENCY | 1,91 | | | 0,02 | |  |  |  |  |  |
| WP254.APOPTOSIS | 1,9 | | | 0,02 | |  |  |  |  |  |
| BIOC_IL7PATHWAY | 1,88 | | | 0,02 | |  |  |  |  |  |
| ARACHIDONIC.ACID.METABOLISM | 1,88 | | | 0,02 | |  |  |  |  |  |
| WP167.EICOSANOID.SYNTHESIS | 1,86 | | | 0,03 | |  |  |  |  |  |
| COMPLEMENT.CASCADE | 1,85 | | | 0,03 | |  |  |  |  |  |
| WP2650.ARACHIDONIC.ACID.METABOLISM | 1,84 | | | 0,03 | |  |  |  |  |  |
| WP2700.LATENT.INFECTION.OF.HOMO.SAPIENS.WITH. MYCOBACTERIUM.TUBERCULOSIS | 1,84 | | | 0,03 | |  |  |  |  |  |
| BIOC_AMIPATHWAY | 1,83 | | | 0,03 | |  |  |  |  |  |
| WP1742.TP53.NETWORK | 1,83 | | | 0,03 | |  |  |  |  |  |
| RECYCLING.OF.BILE.ACIDS.AND.SALTS | 1,82 | | | 0,03 | |  |  |  |  |  |
| WP127.IL.5.SIGNALING.PATHWAY | 1,82 | | | 0,03 | |  |  |  |  |  |
| FC.EPSILON.RECEPTOR.FCERI.SIGNALING | 1,82 | | | 0,03 | |  |  |  |  |  |
| BIOC_CSKPATHWAY | 1,82 | | | 0,03 | |  |  |  |  |  |
| WP1840.INTERLEUKIN.3.5.AND.GM.CSF.SIGNALING | 1,81 | | | 0,03 | |  |  |  |  |  |
| WP2849.HEMATOPOIETIC.STEM.CELL.DIFFERENTIATION | 1,81 | | | 0,03 | |  |  |  |  |  |
| DOWNSTREAM.TCR.SIGNALING | 1,81 | | | 0,03 | |  |  |  |  |  |
| KEGG_GRAFT.VERSUS.HOST.DISEASE | 1,8 | | | 0,03 | |  |  |  |  |  |
| WP22.IL.9.SIGNALING.PATHWAY | 1,8 | | | 0,03 | |  |  |  |  |  |
| WP286.IL.3.SIGNALING.PATHWAY | 1,78 | | | 0,04 | |  |  |  |  |  |
| BINDING.AND.UPTAKE.OF.LIGANDS.BY.SCAVENGER.RECEPTORS | 1,77 | | | 0,04 | |  |  |  |  |  |
| WP1826.GPVI.MEDIATED.ACTIVATION.CASCADE | 1,77 | | | 0,04 | |  |  |  |  |  |
| GENERATION.OF.SECOND.MESSENGER.MOLECULES | 1,77 | | | 0,04 | |  |  |  |  |  |
| SIGNALING.BY.INTERLEUKINS | 1,76 | | | 0,05 | |  |  |  |  |  |
| KEGG_TUBERCULOSIS | 1,75 | | | 0,05 | |  |  |  |  |  |
| WP2507.NANOMATERIAL.INDUCED.APOPTOSIS | 1,75 | | | 0,05 | |  |  |  |  |  |
| PHOSPHORYLATION.OF.CD3.AND.TCR.ZETA.CHAINS | 1,75 | | | 0,05 | |  |  |  |  |  |
| WP2775.TOLL.LIKE.RECEPTORS.CASCADES | 1,73 | | | 0,05 | |  |  |  |  |  |
| INTERLEUKIN.3.5.AND.GM.CSF.SIGNALING | 1,73 | | | 0,06 | |  |  |  |  |  |
| KEGG_STAPHYLOCOCCUS.AUREUS.INFECTION | 1,73 | | | 0,06 | |  |  |  |  |  |
| GPVI.MEDIATED.ACTIVATION.CASCADE | 1,71 | | | 0,06 | |  |  |  |  |  |
| INTERLEUKIN.2.SIGNALING | 1,71 | | | 0,07 | |  |  |  |  |  |
| WP205.IL.7.SIGNALING.PATHWAY | 1,7 | | | 0,07 | |  |  |  |  |  |
| WP1772.APOPTOSIS.MODULATION.AND.SIGNALING | 1,7 | | | 0,07 | |  |  |  |  |  |
| WP2759.FC.EPSILON.RECEPTOR.FCERI.SIGNALING. | 1,69 | | | 0,07 | |  |  |  |  |  |
| BIOC_TOLLPATHWAY | 1,66 | | | 0,09 | |  |  |  |  |  |
| WP2739.AMYLOIDS | 1,65 | | | 0,09 | |  |  |  |  |  |
| KEGG_ARACHIDONIC.ACID.METABOLISM | 1,65 | | | 0,09 | |  |  |  |  |  |
| KEGG_HEMATOPOIETIC.CELL.LINEAGE | 1,65 | | | 0,1 | |  |  |  |  |  |
| LATENT.INFECTION.OF.HOMO.SAPIENS.WITH.MYCOBACTERIUM. TUBERCULOSIS | 1,64 | | | 0,1 | |  |  |  |  |  |
| CHEMOKINE.RECEPTORS.BIND.CHEMOKINES | 1,64 | | | 0,1 | |  |  |  |  |  |
| WP2112.IL17.SIGNALING.PATHWAY | 1,64 | | | 0,1 | |  |  |  |  |  |
| KEGG_CHEMOKINE.SIGNALING.PATHWAY | 1,64 | | | 0,1 | |  |  |  |  |  |
| TOLL.LIKE.RECEPTORS.CASCADES | 1,64 | | | 0,1 | |  |  |  |  |  |
| WP585.INTERFERON.TYPE.I.SIGNALING.PATHWAYS | 1,64 | | | 0,1 | |  |  |  |  |  |
| SYNTHESIS.OF.LEUKOTRIENES.LT.AND.EOXINS.EX. | 1,63 | | | 0,1 | |  |  |  |  |  |
| ROLE.OF.LAT2.NTAL.LAB.ON.CALCIUM.MOBILIZATION | 1,63 | | | 0,1 | |  |  |  |  |  |
| PHAGOSOMAL.MATURATION.EARLY.ENDOSOMAL.STAGE. | 1,63 | | | 0,1 | |  |  |  |  |  |
| BIOC_KERATINOCYTEPATHWAY | 1,63 | | | 0,1 | |  |  |  |  |  |
| WP619.TYPE.II.INTERFERON.SIGNALING.IFNG. | 1,62 | | | 0,1 | |  |  |  |  |  |
| BIOC_CERAMIDEPATHWAY | 1,61 | | | 0,11 | |  |  |  |  |  |
| TRANSLOCATION.OF.ZAP.70.TO.IMMUNOLOGICAL.SYNAPSE | 1,61 | | | 0,11 | |  |  |  |  |  |
| WP1815.FACTORS.INVOLVED.IN.MEGAKARYOCYTE.DEVELOPMENT .AND.PLATELET.PRODUCTION | 1,61 | | | 0,11 | |  |  |  |  |  |
| WP2708.DEGRADATION.OF.COLLAGEN | 1,6 | | | 0,12 | |  |  |  |  |  |
| PHOSPHOLIPID.METABOLISM | 1,59 | | | 0,13 | |  |  |  |  |  |
| PD.1.SIGNALING | 1,59 | | | 0,13 | |  |  |  |  |  |
| TOLL.LIKE.RECEPTOR.4.TLR4.CASCADE | 1,58 | | | 0,12 | |  |  |  |  |  |
| RNA.POLYMERASE.I.PROMOTER.OPENING | 1,58 | | | 0,13 | |  |  |  |  |  |
| WP1433.NOD.PATHWAY | 1,58 | | | 0,13 | |  |  |  |  |  |
| KEGG_LYSOSOME | 1,58 | | | 0,13 | |  |  |  |  |  |
| KEGG_TYPE.I.DIABETES.MELLITUS | 1,57 | | | 0,13 | |  |  |  |  |  |
| WP49.IL.2.SIGNALING.PATHWAY | 1,56 | | | 0,14 | |  |  |  |  |  |
| KEGG_FC.GAMMA.R.MEDIATED.PHAGOCYTOSIS | 1,56 | | | 0,14 | |  |  |  |  |  |
| KEGG_ALLOGRAFT.REJECTION | 1,56 | | | 0,14 | |  |  |  |  |  |
| KEGG_CARBOHYDRATE.DIGESTION.AND.ABSORPTION | 1,55 | | | 0,14 | |  |  |  |  |  |
| BIOC_GLEEVECPATHWAY | 1,55 | | | 0,14 | |  |  |  |  |  |
| KEGG_GLYCEROPHOSPHOLIPID.METABOLISM | 1,54 | | | 0,16 | |  |  |  |  |  |
| TOLL.LIKE.RECEPTOR.TLR6.TLR2.CASCADE | 1,52 | | | 0,17 | |  |  |  |  |  |
| REV.ERBA.REPRESSES.GENE.EXPRESSION | 1,52 | | | 0,17 | |  |  |  |  |  |
| BIOC_BCRPATHWAY | 1,52 | | | 0,17 | |  |  |  |  |  |
| WP2761.MYD88.MAL.CASCADE.INITIATED.ON.PLASMA.MEMBRANE | 1,52 | | | 0,17 | |  |  |  |  |  |
| TOLL.LIKE.RECEPTOR.TLR1.TLR2.CASCADE | 1,52 | | | 0,17 | |  |  |  |  |  |
| SYNTHESIS.OF.IP3.AND.IP4.IN.THE.CYTOSOL | 1,52 | | | 0,17 | |  |  |  |  |  |
| MYD88.MAL.CASCADE.INITIATED.ON.PLASMA.MEMBRANE | 1,51 | | | 0,17 | |  |  |  |  |  |
| WP24.PEPTIDE.GPCRS | 1,51 | | | 0,17 | |  |  |  |  |  |
| KEGG_AUTOIMMUNE.THYROID.DISEASE | 1,51 | | | 0,17 | |  |  |  |  |  |
| WP2447.AMYOTROPHIC.LATERAL.SCLEROSIS.ALS. | 1,51 | | | 0,17 | |  |  |  |  |  |
| TOLL.LIKE.RECEPTOR.2.TLR2.CASCADE | 1,5 | | | 0,18 | |  |  |  |  |  |
| INTERLEUKIN.1.SIGNALING | 1,5 | | | 0,18 | |  |  |  |  |  |
| WP1449.REGULATION.OF.TOLL.LIKE.RECEPTOR.SIGNALING.PATHWAY | 1,5 | | | 0,18 | |  |  |  |  |  |
| RORA.ACTIVATES.CIRCADIAN.GENE.EXPRESSION | 1,5 | | | 0,18 | |  |  |  |  |  |
| KEGG_AFRICAN.TRYPANOSOMIASIS | 1,49 | | | 0,19 | |  |  |  |  |  |
| WP455.GPCRS.CLASS.A.RHODOPSIN.LIKE | 1,49 | | | 0,19 | |  |  |  |  |  |
| BIOC_TCRPATHWAY | 1,49 | | | 0,19 | |  |  |  |  |  |
| WP1904.RIG.I.MDA5.MEDIATED.INDUCTION.OF.IFN.ALPHA.BETA.PATHWAYS | 1,49 | | | 0,19 | |  |  |  |  |  |
| BIOC_STRESSPATHWAY | 1,49 | | | 0,19 | |  |  |  |  |  |
| GLYCEROPHOSPHOLIPID.BIOSYNTHESIS | 1,48 | | | 0,19 | |  |  |  |  |  |
| KEGG_FC.EPSILON.RI.SIGNALING.PATHWAY | 1,48 | | | 0,19 | |  |  |  |  |  |
| DEFENSINS | 1,48 | | | 0,19 | |  |  |  |  |  |
| WP384.APOPTOSIS.MODULATION.BY.HSP70 | 1,48 | | | 0,19 | |  |  |  |  |  |
| WP231.TNF.ALPHA.SIGNALING.PATHWAY | 1,47 | | | 0,19 | |  |  |  |  |  |
| KEGG_INFLAMMATORY.BOWEL.DISEASE.IBD. | 1,47 | | | 0,19 | |  |  |  |  |  |
| WP1799.COSTIMULATION.BY.THE.CD28.FAMILY | 1,47 | | | 0,19 | |  |  |  |  |  |
| WP2203.TSLP.SIGNALING.PATHWAY | 1,47 | | | 0,2 | |  |  |  |  |  |
| WP395.IL.4.SIGNALING.PATHWAY | 1,46 | | | 0,2 | |  |  |  |  |  |
| WP1794.CELL.SURFACE.INTERACTIONS.AT.THE.VASCULAR.WALL | 1,46 | | | 0,2 | |  |  |  |  |  |
| KEGG_LEGIONELLOSIS | 1,46 | | | 0,2 | |  |  |  |  |  |
| WP304.KIT.RECEPTOR.SIGNALING.PATHWAY | 1,46 | | | 0,2 | |  |  |  |  |  |
|  | | | | | | | |  |  |  |
| **Mild intermittent hypoxia exposure - downregulated** | |  | | |  | |  | | | |
| WP2652.MITOTIC.PROMETAPHASE | | -2,46 | | | 0 | |  | | | |
| MITOTIC.PROMETAPHASE | | -2,4 | | | 0 | |  | | | |
| WP466.DNA.REPLICATION | | -2,39 | | | 0 | |  | | | |
| DNA.STRAND.ELONGATION | | -2,36 | | | 0 | |  | | | |
| RESOLUTION.OF.SISTER.CHROMATID.COHESION | | -2,34 | | | 0 | |  | | | |
| KEGG_DNA.REPLICATION | | -2,33 | | | 0 | |  | | | |
| ACTIVATION.OF.ATR.IN.RESPONSE.TO.REPLICATION.STRESS | | -2,31 | | | 0 | |  | | | |
| KEGG_RIBOSOME.BIOGENESIS.IN.EUKARYOTES | | -2,28 | | | 0 | |  | | | |
| WP2446.RB.IN.CANCER | | -2,26 | | | 0 | |  | | | |
| WP2798.ASSEMBLY.OF.COLLAGEN.FIBRILS.AND.OTHER.MULTIMERIC. STRUCTURES | | -2,26 | | | 0 | |  | | | |
| G2.M.CHECKPOINTS | | -2,24 | | | 0 | |  | | | |
| WP2757.MITOTIC.METAPHASE.AND.ANAPHASE | | -2,22 | | | 0 | |  | | | |
| ACTIVATION.OF.THE.PRE.REPLICATIVE.COMPLEX | | -2,21 | | | 0 | |  | | | |
| MITOCHONDRIAL.TRANSLATION | | -2,21 | | | 0 | |  | | | |
| LAMININ.INTERACTIONS | | -2,2 | | | 0 | |  | | | |
| ASSEMBLY.OF.COLLAGEN.FIBRILS.AND.OTHER.MULTIMERIC. STRUCTURES | | -2,2 | | | 0 | |  | | | |
| E2F.MEDIATED.REGULATION.OF.DNA.REPLICATION | | -2,19 | | | 0 | |  | | | |
| CELL.CYCLE.MITOTIC | | -2,19 | | | 0 | |  | | | |
| MITOTIC.M.M.G1.PHASES | | -2,18 | | | 0 | |  | | | |
| MITOCHONDRIAL.TRANSLATION.ELONGATION | | -2,17 | | | 0 | |  | | | |
| MITOCHONDRIAL.TRANSLATION.TERMINATION | | -2,17 | | | 0 | |  | | | |
| DNA.REPAIR | | -2,16 | | | 0 | |  | | | |
| MITOCHONDRIAL.TRANSLATION.INITIATION | | -2,14 | | | 0 | |  | | | |
| SEPARATION.OF.SISTER.CHROMATIDS | | -2,14 | | | 0 | |  | | | |
| MITOTIC.METAPHASE.AND.ANAPHASE | | -2,13 | | | 0 | |  | | | |
| MITOTIC.ANAPHASE | | -2,11 | | | 0 | |  | | | |
| KEGG_MISMATCH.REPAIR | | -2,1 | | | 0 | |  | | | |
| KEGG_CELL.CYCLE | | -2,09 | | | 0 | |  | | | |
| KEGG_AMINOACYL.TRNA.BIOSYNTHESIS | | -2,09 | | | 0 | |  | | | |
| RNA.POLYMERASE.I.PROMOTER.ESCAPE | | -2,08 | | | 0 | |  | | | |
| WP179.CELL.CYCLE | | -2,07 | | | 0 | |  | | | |
| BIOC_VEGFPATHWAY | | -2,07 | | | 0 | |  | | | |
| KEGG_AMINO.SUGAR.AND.NUCLEOTIDE.SUGAR.METABOLISM | | -2,06 | | | 0 | |  | | | |
| KEGG_ECM.RECEPTOR.INTERACTION | | -2,04 | | | 0 | |  | | | |
| WP411.MRNA.PROCESSING | | -2,03 | | | 0 | |  | | | |
| WP1938.TRNA.AMINOACYLATION | | -2,03 | | | 0 | |  | | | |
| TRANSPORT.OF.MATURE.TRANSCRIPT.TO.CYTOPLASM | | -2,02 | | | 0 | |  | | | |
| RNA.POLYMERASE.II.TRANSCRIPTION | | -2,02 | | | 0 | |  | | | |
| LAGGING.STRAND.SYNTHESIS | | -2,01 | | | 0 | |  | | | |
| WP2006.MIR.TARGETED.GENES.IN.SQUAMOUS.CELL.TARBASE | | -2,01 | | | 0 | |  | | | |
| TRANSPORT.OF.MATURE.MRNA.DERIVED.FROM.AN.INTRON. CONTAINING.TRANSCRIPT | | -2,01 | | | 0 | |  | | | |
| PHOSPHORYLATION.OF.THE.APC.C | | -2,01 | | | 0 | |  | | | |
| WP2715.METABOLISM.OF.NON.CODING.RNA | | -2 | | | 0 | |  | | | |
| TRANSCRIPTION.OF.THE.HIV.GENOME | | -2 | | | 0 | |  | | | |
| EXTENSION.OF.TELOMERES | | -1,99 | | | 0 | |  | | | |
| WP1785.ASPARAGINE.N.LINKED.GLYCOSYLATION | | -1,99 | | | 0 | |  | | | |
| INACTIVATION.OF.APC.C.VIA.DIRECT.INHIBITION.OF.THE.APC.C. COMPLEX | | -1,99 | | | 0 | |  | | | |
| TRNA.AMINOACYLATION | | -1,99 | | | 0 | |  | | | |
| WP1889.PROCESSING.OF.CAPPED.INTRON.CONTAINING.PRE.MRNA | | -1,98 | | | 0 | |  | | | |
| RNA.POLYMERASE.II.PRE.TRANSCRIPTION.EVENTS | | -1,98 | | | 0 | |  | | | |
| MITOTIC.SPINDLE.CHECKPOINT | | -1,98 | | | 0 | |  | | | |
| XBP1.S.ACTIVATES.CHAPERONE.GENES | | -1,97 | | | 0 | |  | | | |
| WP1807.DOUBLE.STRAND.BREAK.REPAIR | | -1,97 | | | 0 | |  | | | |
| FORMATION.OF.TUBULIN.FOLDING.INTERMEDIATES.BY.CCT.TRIC | | -1,97 | | | 0 | |  | | | |
| IRE1ALPHA.ACTIVATES.CHAPERONES | | -1,96 | | | 0 | |  | | | |
| METABOLISM.OF.NON.CODING.RNA | | -1,96 | | | 0 | |  | | | |
| M.PHASE | | -1,96 | | | 0 | |  | | | |
| CELL.CYCLE.CHECKPOINTS | | -1,96 | | | 0,01 | |  | | | |
| INHIBITION.OF.THE.PROTEOLYTIC.ACTIVITY.OF.APC.C.REQUIRED. FOR.THE.ONSET.OF.ANAPHASE.BY.MITOTIC.SPINDLE.CHECKPOINT. COMPONENTS | | -1,96 | | | 0 | |  | | | |
| G1.S.TRANSITION | | -1,95 | | | 0,01 | |  | | | |
| RNA.POLYMERASE.II.TRANSCRIPTION.ELONGATION | | -1,95 | | | 0,01 | |  | | | |
| PROCESSING.OF.CAPPED.INTRON.CONTAINING.PRE.MRNA | | -1,95 | | | 0,01 | |  | | | |
| RNA.POLYMERASE.I.TRANSCRIPTION.INITIATION | | -1,94 | | | 0,01 | |  | | | |
| WP1980.NUCLEOTIDE.EXCISION.REPAIR | | -1,94 | | | 0,01 | |  | | | |
| DNA.REPLICATION | | -1,94 | | | 0,01 | |  | | | |
| S.PHASE | | -1,94 | | | 0,01 | |  | | | |
| SNRNP.ASSEMBLY | | -1,93 | | | 0,01 | |  | | | |
| SMOOTH.MUSCLE.CONTRACTION | | -1,93 | | | 0,01 | |  | | | |
| UNFOLDED.PROTEIN.RESPONSE.UPR. | | -1,93 | | | 0,01 | |  | | | |
| RNA.POLYMERASE.I.TRANSCRIPTION.TERMINATION | | -1,93 | | | 0,01 | |  | | | |
| COLLAGEN.FORMATION | | -1,93 | | | 0,01 | |  | | | |
| WP2667.ACTIVATION.OF.CHAPERONE.GENES.BY.XBP1.S. | | -1,92 | | | 0,01 | |  | | | |
| WP1859.MITOTIC.G2.G2.M.PHASES | | -1,92 | | | 0,01 | |  | | | |
| PREFOLDIN.MEDIATED.TRANSFER.OF.SUBSTRATE.TO.CCT.TRIC | | -1,92 | | | 0,01 | |  | | | |
| WP2772.S.PHASE | | -1,91 | | | 0,01 | |  | | | |
| MITOTIC.G2.G2.M.PHASES | | -1,91 | | | 0,01 | |  | | | |
| RNA.POLYMERASE.II.TRANSCRIPTION.INITIATION.AND.PROMOTER. CLEARANCE | | -1,91 | | | 0,01 | |  | | | |
| SIGNALING.BY.BMP | | -1,91 | | | 0,01 | |  | | | |
| LATE.PHASE.OF.HIV.LIFE.CYCLE | | -1,91 | | | 0,01 | |  | | | |
| RNA.POLYMERASE.II.PROMOTER.ESCAPE | | -1,91 | | | 0,01 | |  | | | |
| RNA.POLYMERASE.II.TRANSCRIPTION.PRE.INITIATION.AND.PROMOTER. OPENING | | -1,91 | | | 0,01 | |  | | | |
| FORMATION.OF.RNA.POL.II.ELONGATION.COMPLEX | | -1,91 | | | 0,01 | |  | | | |
| TAT.MEDIATED.ELONGATION.OF.THE.HIV.1.TRANSCRIPT | | -1,91 | | | 0,01 | |  | | | |
| G2.M.TRANSITION | | -1,91 | | | 0,01 | |  | | | |
| CYTOSOLIC.TRNA.AMINOACYLATION | | -1,9 | | | 0,01 | |  | | | |
| POST.CHAPERONIN.TUBULIN.FOLDING.PATHWAY | | -1,9 | | | 0,01 | |  | | | |
| MRNA.CAPPING | | -1,9 | | | 0,01 | |  | | | |
| G1.S.SPECIFIC.TRANSCRIPTION | | -1,9 | | | 0,01 | |  | | | |
| RNA.POLYMERASE.II.HIV.PROMOTER.ESCAPE | | -1,9 | | | 0,01 | |  | | | |
| CYCLIN.A.B1.ASSOCIATED.EVENTS.DURING.G2.M.TRANSITION | | -1,9 | | | 0,01 | |  | | | |
| WP1861.MRNA.CAPPING | | -1,9 | | | 0,01 | |  | | | |
| HIV.TRANSCRIPTION.INITIATION | | -1,9 | | | 0,01 | |  | | | |
| RESPIRATORY.ELECTRON.TRANSPORT | | -1,9 | | | 0,01 | |  | | | |
| FORMATION.OF.HIV.ELONGATION.COMPLEX.IN.THE. ABSENCE.OF.HIV.TAT | | -1,89 | | | 0,01 | |  | | | |
| TRANSCRIPTION.COUPLED.NER.TC.NER. | | -1,89 | | | 0,01 | |  | | | |
| FORMATION.OF.HIV.1.ELONGATION.COMPLEX.CONTAINING. HIV.1.TAT | | -1,89 | | | 0,01 | |  | | | |
| O.GLYCOSYLATION.OF.TSR.DOMAIN.CONTAINING.PROTEINS | | -1,88 | | | 0,01 | |  | | | |
| WP1775.CELL.CYCLE.CHECKPOINTS | | -1,88 | | | 0,01 | |  | | | |
| RNA.POLYMERASE.II.TRANSCRIPTION.INITIATION | | -1,88 | | | 0,01 | |  | | | |
| KEGG_PROTEIN.EXPORT | | -1,88 | | | 0,01 | |  | | | |
| WP405.EUKARYOTIC.TRANSCRIPTION.INITIATION | | -1,87 | | | 0,01 | |  | | | |
| MRNA.3.END.PROCESSING | | -1,87 | | | 0,01 | |  | | | |
| INTERACTIONS.OF.REV.WITH.HOST.CELLULAR.PROTEINS | | -1,87 | | | 0,01 | |  | | | |
| NUCLEOTIDE.EXCISION.REPAIR | | -1,86 | | | 0,01 | |  | | | |
| MRNA.SPLICING.MAJOR.PATHWAY | | -1,86 | | | 0,01 | |  | | | |
| POST.ELONGATION.PROCESSING.OF.INTRON. CONTAINING.PRE.MRNA | | -1,86 | | | 0,01 | |  | | | |
| KEGG_NUCLEOTIDE.EXCISION.REPAIR | | -1,86 | | | 0,01 | |  | | | |
| TRANSPORT.OF.THE.SLBP.INDEPENDENT.MATURE.MRNA | | -1,85 | | | 0,01 | |  | | | |
| WP1906.RNA.POLYMERASE.II.TRANSCRIPTION | | -1,85 | | | 0,01 | |  | | | |
| HIV.TRANSCRIPTION.ELONGATION | | -1,85 | | | 0,01 | |  | | | |
| REV.MEDIATED.NUCLEAR.EXPORT.OF.HIV.RNA | | -1,85 | | | 0,01 | |  | | | |
| COOPERATION.OF.PREFOLDIN.AND.TRIC.CCT.IN.ACTIN. AND.TUBULIN.FOLDING | | -1,85 | | | 0,01 | |  | | | |
| SYNTHESIS.OF.DNA | | -1,85 | | | 0,01 | |  | | | |
| MRNA.SPLICING | | -1,85 | | | 0,01 | |  | | | |
| DOUBLE.STRAND.BREAK.REPAIR | | -1,85 | | | 0,01 | |  | | | |
| TELOMERE.C.STRAND.LAGGING.STRAND.SYNTHESIS | | -1,85 | | | 0,01 | |  | | | |
| WP2005.MIR.TARGETED.GENES.IN.MUSCLE.CELL.TARBASE | | -1,85 | | | 0,01 | |  | | | |
| WP1925.SYNTHESIS.OF.DNA | | -1,84 | | | 0,01 | |  | | | |
| HIV.LIFE.CYCLE | | -1,84 | | | 0,01 | |  | | | |
| COLLAGEN.BIOSYNTHESIS.AND.MODIFYING.ENZYMES | | -1,84 | | | 0,01 | |  | | | |
| WP2654.MITOTIC.PROPHASE | | -1,83 | | | 0,01 | |  | | | |
| TRANSCRIPTION | | -1,83 | | | 0,01 | |  | | | |
| FANCONI.ANEMIA.PATHWAY | | -1,83 | | | 0,01 | |  | | | |
| MITOCHONDRIAL.TRNA.AMINOACYLATION | | -1,83 | | | 0,01 | |  | | | |
| ELASTIC.FIBRE.FORMATION | | -1,81 | | | 0,01 | |  | | | |
| TRANSPORT.OF.THE.SLBP.DEPENDANT.MATURE.MRNA | | -1,81 | | | 0,01 | |  | | | |
| PROTEIN.FOLDING | | -1,8 | | | 0,01 | |  | | | |
| EXTRACELLULAR.MATRIX.ORGANIZATION | | -1,79 | | | 0,02 | |  | | | |
| BIOC_PROTEASOMEPATHWAY | | -1,79 | | | 0,02 | |  | | | |
| NUCLEAR.IMPORT.OF.REV.PROTEIN | | -1,79 | | | 0,02 | |  | | | |
| ORGANELLE.BIOGENESIS.AND.MAINTENANCE | | -1,79 | | | 0,02 | |  | | | |
| KEGG_SPLICEOSOME | | -1,78 | | | 0,02 | |  | | | |
| INTERACTIONS.OF.VPR.WITH.HOST.CELLULAR.PROTEINS | | -1,78 | | | 0,02 | |  | | | |
| KEGG_FANCONI.ANEMIA.PATHWAY | | -1,78 | | | 0,02 | |  | | | |
| CHROMOSOME.MAINTENANCE | | -1,77 | | | 0,02 | |  | | | |
| GLOBAL.GENOMIC.NER.GG.NER. | | -1,77 | | | 0,02 | |  | | | |
| TRANSPORT.OF.MATURE.MRNAS.DERIVED.FROM. INTRONLESS.TRANSCRIPTS | | -1,77 | | | 0,02 | |  | | | |
| MITOTIC.G1.G1.S.PHASES | | -1,76 | | | 0,02 | |  | | | |
| WP2004.MIR.TARGETED.GENES.IN.LYMPHOCYTES.TARBASE | | -1,76 | | | 0,02 | |  | | | |
| KEGG_RNA.TRANSPORT | | -1,75 | | | 0,02 | |  | | | |
| CENTROSOME.MATURATION | | -1,75 | | | 0,02 | |  | | | |
| RECRUITMENT.OF.MITOTIC.CENTROSOME.PROTEINS. AND.COMPLEXES | | -1,75 | | | 0,02 | |  | | | |
| EPIGENETIC.REGULATION.OF.GENE.EXPRESSION | | -1,75 | | | 0,02 | |  | | | |
| WP2760.SIGNALING.BY.BMP | | -1,74 | | | 0,02 | |  | | | |
| KEGG_BASAL.TRANSCRIPTION.FACTORS | | -1,74 | | | 0,02 | |  | | | |
| KEGG_PURINE.METABOLISM | | -1,74 | | | 0,02 | |  | | | |
| GAP.FILLING.DNA.REPAIR.SYNTHESIS.AND.LIGATION. IN.GG.NER | | -1,74 | | | 0,02 | |  | | | |
| WP1892.PROTEIN.FOLDING | | -1,74 | | | 0,02 | |  | | | |
| NUCLEAR.PORE.COMPLEX.NPC.DISASSEMBLY | | -1,74 | | | 0,02 | |  | | | |
| HOMOLOGOUS.RECOMBINATION.REPAIR | | -1,74 | | | 0,02 | |  | | | |
| REPAIR.SYNTHESIS.OF.PATCH.27.30.BASES.LONG.BY. DNA.POLYMERASE | | -1,73 | | | 0,02 | |  | | | |
| HOMOLOGOUS.RECOMBINATION.REPAIR.OF.REPLICATION .INDEPENDENT.DOUBLE.STRAND.BREAKS | | -1,73 | | | 0,03 | |  | | | |
| NON.INTEGRIN.MEMBRANE.ECM.INTERACTIONS | | -1,72 | | | 0,03 | |  | | | |
| SCAVENGING.BY.CLASS.A.RECEPTORS | | -1,72 | | | 0,03 | |  | | | |
| RNA.POL.II.CTD.PHOSPHORYLATION.AND.INTERACTION. WITH.CE | | -1,72 | | | 0,03 | |  | | | |
| MRNA.SPLICING.MINOR.PATHWAY | | -1,72 | | | 0,03 | |  | | | |
| WP1858.MITOTIC.G1.G1.S.PHASES | | -1,71 | | | 0,03 | |  | | | |
| M.G1.TRANSITION | | -1,71 | | | 0,03 | |  | | | |
| G0.AND.EARLY.G1 | | -1,71 | | | 0,03 | |  | | | |
| KEGG_OOCYTE.MEIOSIS | | -1,71 | | | 0,03 | |  | | | |
| WP1928.TELOMERE.MAINTENANCE | | -1,71 | | | 0,03 | |  | | | |
| KEGG_SELENOCOMPOUND.METABOLISM | | -1,71 | | | 0,03 | |  | | | |
| REPAIR.SYNTHESIS.FOR.GAP.FILLING.BY.DNA. POLYMERASE.IN.TC.NER | | -1,71 | | | 0,03 | |  | | | |
| WP2658.HIV.LIFE.CYCLE | | -1,71 | | | 0,03 | |  | | | |
| CONVERSION.FROM.APC.C.CDC20.TO.APC.C.CDH1.IN. LATE.ANAPHASE | | -1,71 | | | 0,03 | |  | | | |
| WP2002.MIR.TARGETED.GENES.IN.EPITHELIUM.TARBASE | | -1,71 | | | 0,03 | |  | | | |
| WP2785.M.G1.TRANSITION | | -1,7 | | | 0,03 | |  | | | |
| DNA.REPLICATION.PRE.INITIATION | | -1,7 | | | 0,03 | |  | | | |
| WP2672.ISG15.ANTIVIRAL.MECHANISM | | -1,7 | | | 0,03 | |  | | | |
| FORMATION.OF.TRANSCRIPTION.COUPLED.NER.TC.NER.REPAIR.COMPLEX | | -1,7 | | | 0,03 | |  | | | |
| TRANSPORT.OF.MATURE.MRNA.DERIVED.FROM.AN.INTRONLESS.TRANSCRIPT | | -1,7 | | | 0,03 | |  | | | |
| WP2679.MHC.CLASS.II.ANTIGEN.PRESENTATION | | -1,7 | | | 0,03 | |  | | | |
| RESPIRATORY.ELECTRON.TRANSPORT.ATP.SYNTHESIS.BY.CHEMIOSMOTIC. COUPLING.AND.HEAT.PRODUCTION.BY.UNCOUPLING.PROTEINS. | | -1,7 | | | 0,03 | |  | | | |
| KEGG_ONE.CARBON.POOL.BY.FOLATE | | -1,7 | | | 0,03 | |  | | | |
| APC.CDC20.MEDIATED.DEGRADATION.OF.NEK2A | | -1,69 | | | 0,03 | |  | | | |
| WP1816.FANCONI.ANEMIA.PATHWAY | | -1,69 | | | 0,03 | |  | | | |
| WP111.ELECTRON.TRANSPORT.CHAIN | | -1,69 | | | 0,03 | |  | | | |
| FORMATION.OF.THE.EARLY.ELONGATION.COMPLEX | | -1,69 | | | 0,03 | |  | | | |
| CGMP.EFFECTS | | -1,69 | | | 0,03 | |  | | | |
| PURINE.METABOLISM | | -1,68 | | | 0,03 | |  | | | |
| GAP.FILLING.DNA.REPAIR.SYNTHESIS.AND.LIGATION.IN.TC.NER | | -1,68 | | | 0,03 | |  | | | |
| WP1902.RESPIRATORY.ELECTRON.TRANSPORT.ATP.SYNTHESIS.BY. CHEMIOSMOTIC.COUPLING.AND.HEAT.PRODUCTION.BY.UNCOUPLING. PROTEINS. | | -1,68 | | | 0,03 | |  | | | |
| VPR.MEDIATED.NUCLEAR.IMPORT.OF.PICS | | -1,68 | | | 0,03 | |  | | | |
| MOLECULES.ASSOCIATED.WITH.ELASTIC.FIBRES | | -1,68 | | | 0,03 | |  | | | |
| DUAL.INCISION.REACTION.IN.TC.NER | | -1,68 | | | 0,03 | |  | | | |
| BIOC_NO1PATHWAY | | -1,67 | | | 0,03 | |  | | | |
| KEGG_FOCAL.ADHESION | | -1,67 | | | 0,03 | |  | | | |
| CLEAVAGE.OF.GROWING.TRANSCRIPT.IN.THE.TERMINATION.REGION | | -1,66 | | | 0,04 | |  | | | |
| REGULATION.OF.GLUCOKINASE.BY.GLUCOKINASE.REGULATORY. PROTEIN | | -1,66 | | | 0,04 | |  | | | |
| RNA.POLYMERASE.II.TRANSCRIPTION.TERMINATION | | -1,66 | | | 0,04 | |  | | | |
| REGULATION.OF.PLK1.ACTIVITY.AT.G2.M.TRANSITION | | -1,66 | | | 0,04 | |  | | | |
| REGULATION.OF.MITOTIC.CELL.CYCLE | | -1,65 | | | 0,04 | |  | | | |
| WP2725.COLLAGEN.BIOSYNTHESIS.AND.MODIFYING.ENZYMES | | -1,65 | | | 0,04 | |  | | | |
| POST.TRANSLATIONAL.PROTEIN.MODIFICATION | | -1,65 | | | 0,04 | |  | | | |
| APC.C.CDC20.MEDIATED.DEGRADATION.OF.CYCLIN.B | | -1,64 | | | 0,04 | |  | | | |
| LOSS.OF.PROTEINS.REQUIRED.FOR.INTERPHASE.MICROTUBULE. ORGANIZATION.FROM.THE.CENTROSOME | | -1,64 | | | 0,04 | |  | | | |
| LOSS.OF.NLP.FROM.MITOTIC.CENTROSOMES | | -1,64 | | | 0,04 | |  | | | |
| POST.ELONGATION.PROCESSING.OF.THE.TRANSCRIPT | | -1,64 | | | 0,04 | |  | | | |
| PROCESSIVE.SYNTHESIS.ON.THE.LAGGING.STRAND | | -1,64 | | | 0,04 | |  | | | |
| APC.C.MEDIATED.DEGRADATION.OF.CELL.CYCLE. PROTEINS | | -1,63 | | | 0,04 | |  | | | |
| CHAPERONIN.MEDIATED.PROTEIN.FOLDING | | -1,63 | | | 0,04 | |  | | | |
| FORMATION.OF.THE.HIV.1.EARLY.ELONGATION.COMPLEX | | -1,63 | | | 0,05 | |  | | | |
| MISMATCH.REPAIR.MMR.DIRECTED.BY.MSH2.MSH6. MUTSALPHA. | | -1,62 | | | 0,05 | |  | | | |
| MISMATCH.REPAIR | | -1,62 | | | 0,05 | |  | | | |
| WP1782.APC.C.MEDIATED.DEGRADATION.OF.CELL.CYCLE. PROTEINS | | -1,61 | | | 0,05 | |  | | | |
| WP45.G1.TO.S.CELL.CYCLE.CONTROL | | -1,61 | | | 0,05 | |  | | | |
| WP1873.NGF.SIGNALLING.VIA.TRKA.FROM.THE.PLASMA. MEMBRANE | | -1,61 | | | 0,05 | |  | | | |
| EPH.EPHRIN.SIGNALING | | -1,61 | | | 0,05 | |  | | | |
| KEGG_PROTEIN.PROCESSING.IN.ENDOPLASMIC.RETICULUM | | -1,59 | | | 0,06 | |  | | | |
| KEGG_PYRIMIDINE.METABOLISM | | -1,59 | | | 0,06 | |  | | | |
| WP2703.EXTRACELLULAR.MATRIX.ORGANIZATION | | -1,59 | | | 0,06 | |  | | | |
| GENERIC.TRANSCRIPTION.PATHWAY | | -1,59 | | | 0,06 | |  | | | |
| REGULATION.OF.APC.C.ACTIVATORS.BETWEEN.G1.S.AND. EARLY.ANAPHASE | | -1,59 | | | 0,06 | |  | | | |
| KEGG_PI3K.AKT.SIGNALING.PATHWAY | | -1,59 | | | 0,06 | |  | | | |
| WP244.ALPHA.6.BETA.4.SIGNALING.PATHWAY | | -1,58 | | | 0,06 | |  | | | |
| EPHB.MEDIATED.FORWARD.SIGNALING | | -1,58 | | | 0,06 | |  | | | |
| ASPARAGINE.N.LINKED.GLYCOSYLATION | | -1,58 | | | 0,06 | |  | | | |
| BIOC_ERYTHPATHWAY | | -1,57 | | | 0,07 | |  | | | |
| BIOC_EDG1PATHWAY | | -1,57 | | | 0,07 | |  | | | |
| TIE2.SIGNALING | | -1,57 | | | 0,07 | |  | | | |
| WP1539.ANGIOGENESIS | | -1,56 | | | 0,07 | |  | | | |
| MISMATCH.REPAIR.MMR.DIRECTED.BY.MSH2.MSH3.MUTSBETA. | | -1,56 | | | 0,07 | |  | | | |
| BIOC_ARFPATHWAY | | -1,56 | | | 0,07 | |  | | | |
| NEP.NS2.INTERACTS.WITH.THE.CELLULAR.EXPORT. MACHINERY | | -1,56 | | | 0,07 | |  | | | |
| NEGATIVE.EPIGENETIC.REGULATION.OF.RRNA. EXPRESSION | | -1,56 | | | 0,07 | |  | | | |
| BIOC_MTORPATHWAY | | -1,56 | | | 0,07 | |  | | | |
| WP241.ONE.CARBON.METABOLISM | | -1,55 | | | 0,07 | |  | | | |
| NUCLEAR.ENVELOPE.BREAKDOWN | | -1,55 | | | 0,08 | |  | | | |
| ECM.PROTEOGLYCANS | | -1,54 | | | 0,08 | |  | | | |
| NITRIC.OXIDE.STIMULATES.GUANYLATE.CYCLASE | | -1,53 | | | 0,09 | |  | | | |
| RNA.POLYMERASE.I.RNA.POLYMERASE.III.AND. MITOCHONDRIAL.TRANSCRIPTION | | -1,53 | | | 0,09 | |  | | | |
| ACTIVATION.OF.APC.C.AND.APC.C.CDC20.MEDIATED. DEGRADATION.OF.MITOTIC.PROTEINS | | -1,52 | | | 0,09 | |  | | | |
| KEGG_GAP.JUNCTION | | -1,52 | | | 0,09 | |  | | | |
| GLUTAMATE.NEUROTRANSMITTER.RELEASE.CYCLE | | -1,51 | | | 0,1 | |  | | | |
| NORC.NEGATIVELY.REGULATES.RRNA.EXPRESSION | | -1,51 | | | 0,1 | |  | | | |
| WP2784.BINDING.AND.UPTAKE.OF.LIGANDS.BY.SCAVENGER. RECEPTORS | | -1,51 | | | 0,1 | |  | | | |
| WP1905.RNA.POLYMERASE.I.RNA.POLYMERASE.III.AND. MITOCHONDRIAL.TRANSCRIPTION | | -1,51 | | | 0,1 | |  | | | |
| SIGNALING.BY.FGFR2.MUTANTS | | -1,51 | | | 0,1 | |  | | | |
| METABOLISM.OF.NUCLEOTIDES | | -1,5 | | | 0,11 | |  | | | |
| ASSOCIATION.OF.TRIC.CCT.WITH.TARGET.PROTEINS.DURING. BIOSYNTHESIS | | -1,49 | | | 0,11 | |  | | | |
| BIOC_INFLAMPATHWAY | | -1,49 | | | 0,11 | |  | | | |
| EXPORT.OF.VIRAL.RIBONUCLEOPROTEINS.FROM.NUCLEUS | | -1,48 | | | 0,12 | |  | | | |
| EPHA.MEDIATED.GROWTH.CONE.COLLAPSE | | -1,48 | | | 0,12 | |  | | | |
| REMOVAL.OF.LICENSING.FACTORS.FROM.ORIGINS | | -1,48 | | | 0,12 | |  | | | |
| ISG15.ANTIVIRAL.MECHANISM | | -1,48 | | | 0,12 | |  | | | |
| HIV.INFECTION | | -1,48 | | | 0,12 | |  | | | |
| APC.C.CDC20.MEDIATED.DEGRADATION.OF.MITOTIC.PROTEINS | | -1,47 | | | 0,12 | |  | | | |
| WP2363.GASTRIC.CANCER.NETWORK.2 | | -1,47 | | | 0,13 | |  | | | |
| KEGG_DILATED.CARDIOMYOPATHY | | -1,47 | | | 0,13 | |  | | | |
| REGULATION.OF.DNA.REPLICATION | | -1,46 | | | 0,13 | |  | | | |
| TELOMERE.MAINTENANCE | | -1,46 | | | 0,13 | |  | | | |
| WP1885.PLATELET.HOMEOSTASIS | | -1,46 | | | 0,13 | |  | | | |
| MITOTIC.PROPHASE | | -1,46 | | | 0,13 | |  | | | |
| ANTIVIRAL.MECHANISM.BY.IFN.STIMULATED.GENES | | -1,46 | | | 0,13 | |  | | | |
| WP107.TRANSLATION.FACTORS | | -1,45 | | | 0,14 | |  | | | |
| KEGG_PROGESTERONE.MEDIATED.OOCYTE.MATURATION | | -1,45 | | | 0,14 | |  | | | |
| AXON.GUIDANCE | | -1,44 | | | 0,14 | |  | | | |
| WP1898.REGULATION.OF.DNA.REPLICATION | | -1,44 | | | 0,15 | |  | | | |
| TRANSLATION | | -1,44 | | | 0,15 | |  | | | |
| WP2683.INFLUENZA.LIFE.CYCLE | | -1,43 | | | 0,15 | |  | | | |
| SIGNALING.BY.HEDGEHOG | | -1,43 | | | 0,15 | |  | | | |
| HEDGEHOG.OFF.STATE | | -1,42 | | | 0,16 | |  | | | |
| KEGG_VASCULAR.SMOOTH.MUSCLE.CONTRACTION | | -1,42 | | | 0,16 | |  | | | |
| O.LINKED.GLYCOSYLATION | | -1,42 | | | 0,17 | |  | | | |
| RNA.POLYMERASE.I.TRANSCRIPTION | | -1,41 | | | 0,17 | |  | | | |
| AUTODEGRADATION.OF.CDH1.BY.CDH1.APC.C | | -1,41 | | | 0,17 | |  | | | |
| KEGG_PROTEIN.DIGESTION.AND.ABSORPTION | | -1,4 | | | 0,18 | |  | | | |
| CDC20.PHOSPHO.APC.C.MEDIATED.DEGRADATION.OF.CYCLIN.A | | -1,4 | | | 0,18 | |  | | | |
| MEIOSIS | | -1,4 | | | 0,18 | |  | | | |
| APC.C.CDH1.MEDIATED.DEGRADATION.OF.CDC20.AND.OTHER. APC.C.CDH1.TARGETED.PROTEINS.IN.LATE.MITOSIS.EARLY.G1 | | -1,39 | | | 0,18 | |  | | | |
| SIGNALING.BY.VEGF | | -1,38 | | | 0,19 | |  | | | |
| WP306.FOCAL.ADHESION | | -1,38 | | | 0,19 | |  | | | |

### **Supplementary Table 2.** Top 100 genes significantly upregulated (positive values) and downregulated (negative values) by mild intermittent hypoxia compared to normoxia exposure in human skeletal muscle (*m. vastus lateralis*) (*n*=11).

| **Gene symbol** | **Gene name** | **Entrez ID** | **Mean fold change** | **IBMT regularized paired t-test raw p-value** |
| --- | --- | --- | --- | --- |
| **Mild intermittent hypoxia exposure - upregulation** | | | | |
| CORT | Cortistatin | 1325 | 2,37 | 5,10E-05 |
| APOC1 | apolipoprotein C-I | 341 | 1,9 | 3,10E-04 |
| LINC01194 | long intergenic non-protein coding RNA 1194 | 404663 | 1,9 | 7,30E-03 |
| SPIN2B | spindlin family, member 2B | 474343 | 1,81 | 3,70E-03 |
| LOC101928236 | uncharacterized LOC101928236 | 101928236 | 1,74 | 5,10E-03 |
| CXCL1 | chemokine (C-X-C motif) ligand 1 (melanoma growth stimulating activity, alpha) | 2919 | 1,73 | 7,90E-03 |
| SNHG4 | small nucleolar RNA host gene 4 (non-protein coding) | 724102 | 1,71 | 5,10E-03 |
| C2orf27A | chromosome 2 open reading frame 27A | 29798 | 1,69 | 1,30E-02 |
| LCN2 | lipocalin 2 | 3934 | 1,69 | 3,30E-04 |
| OR2AT4 | olfactory receptor, family 2, subfamily AT, member 4 | 341152 | 1,68 | 1,30E-02 |
| TCTEX1D2 | Tctex1 domain containing 2 | 255758 | 1,67 | 6,30E-03 |
| S100A7A | S100 calcium binding protein A7A | 338324 | 1,64 | 4,10E-03 |
| GSTM5 | glutathione S-transferase mu 5 | 2949 | 1,6 | 6,40E-03 |
| ARHGEF33 | Rho guanine nucleotide exchange factor (GEF) 33 | 100271715 | 1,59 | 1,40E-04 |
| GOLGA8DP | golgin A8 family, member D, pseudogene | 100132979 | 1,58 | 1,80E-03 |
| SCGB3A1 | secretoglobin, family 3A, member 1 | 92304 | 1,57 | 2,30E-03 |
| ZNF285 | zinc finger protein 285 | 26974 | 1,54 | 3,70E-03 |
| LOC283683 | uncharacterized LOC283683 | 283683 | 1,54 | 1,10E-03 |
| LOC101927973 | uncharacterized LOC101927973 | 101927973 | 1,54 | 1,00E-02 |
| LINC01534 | long intergenic non-protein coding RNA 1534 | 101927621 | 1,51 | 7,60E-03 |
| EXTL3-AS1 | EXTL3 antisense RNA 1 | 101929402 | 1,51 | 1,40E-02 |
| LINC00263 | long intergenic non-protein coding RNA 263 | 90271 | 1,51 | 3,70E-02 |
| ACOT1 | acyl-CoA thioesterase 1 | 641371 | 1,5 | 2,30E-02 |
| SH3GL1 | SH3-domain GRB2-like 1 | 6455 | 1,49 | 3,70E-03 |
| LOC100131289 | uncharacterized LOC100131289 | 100131289 | 1,48 | 2,40E-02 |
| WASH1 | WAS protein family homolog 1 | 100287171 | 1,48 | 1,00E-02 |
| MIR3192 | microRNA 3192 | 100422875 | 1,47 | 2,70E-02 |
| FAM179A | family with sequence similarity 179, member A | 165186 | 1,47 | 3,20E-03 |
| LOC100507387 | uncharacterized LOC100507387 | 100507387 | 1,47 | 8,90E-03 |
| SLC5A6 | solute carrier family 5 (sodium/multivitamin and iodide cotransporter), member 6 | 8884 | 1,47 | 1,60E-02 |
| LOC101927696 | uncharacterized LOC101927696 | 101927696 | 1,47 | 2,00E-02 |
| LOC146880 | Rho GTPase activating protein 27 pseudogene | 146880 | 1,45 | 7,30E-03 |
| CHGB | chromogranin B (secretogranin 1) | 1114 | 1,45 | 4,40E-03 |
| ZNF613 | zinc finger protein 613 | 79898 | 1,45 | 1,40E-02 |
| CAPG | capping protein (actin filament), gelsolin-like | 822 | 1,45 | 1,30E-02 |
| MYZAP | myocardial zonula adherens protein | 100820829 | 1,44 | 2,50E-02 |
| IPO5P1 | importin 5 pseudogene 1 | 100132815 | 1,44 | 1,90E-02 |
| TRAPPC2 | trafficking protein particle complex 2 | 6399 | 1,44 | 4,30E-03 |
| OSR1 | odd-skipped related transciption factor 1 | 130497 | 1,44 | 6,50E-03 |
| LY6G5C | lymphocyte antigen 6 complex, locus G5C | 80741 | 1,44 | 3,40E-02 |
| TRMT112 | tRNA methyltransferase 11-2 homolog (S. cerevisiae) | 51504 | 1,44 | 1,40E-02 |
| TRAJ35 | T cell receptor alpha joining 35 (non-functional) | 28720 | 1,44 | 2,90E-02 |
| CASC22 | cancer susceptibility candidate 22 (non-protein coding) | 283854 | 1,44 | 4,40E-02 |
| QPCT | glutaminyl-peptide cyclotransferase | 25797 | 1,43 | 1,20E-02 |
| TRIM16L | tripartite motif containing 16-like | 147166 | 1,43 | 1,50E-02 |
| LOC100506368 | uncharacterized LOC100506368 | 100506368 | 1,43 | 1,90E-02 |
| LOC101929592 | uncharacterized LOC101929592 | 101929592 | 1,43 | 6,30E-03 |
| OR2L8 | olfactory receptor, family 2, subfamily L, member 8 | 391190 | 1,43 | 7,20E-03 |
| CAMKMT | calmodulin-lysine N-methyltransferase | 79823 | 1,42 | 1,30E-02 |
| ARPIN | actin-related protein 2/3 complex inhibitor | 348110 | 1,42 | 3,20E-02 |
| OR2A5 | olfactory receptor, family 2, subfamily A, member 5 | 393046 | 1,42 | 1,20E-02 |
| FCGBP | Fc fragment of IgG binding protein | 8857 | 1,42 | 3,90E-03 |
| UBXN8 | UBX domain protein 8 | 7993 | 1,42 | 1,70E-02 |
| MIR3622B | microRNA 3622b | 100500871 | 1,41 | 1,60E-02 |
| FGF13-AS1 | FGF13 antisense RNA 1 | 100129662 | 1,41 | 3,20E-02 |
| SCARNA15 | small Cajal body-specific RNA 15 | 677778 | 1,41 | 1,30E-02 |
| BMP1 | bone morphogenetic protein 1 | 649 | 1,4 | 3,80E-02 |
| SLC13A3 | solute carrier family 13 (sodium-dependent dicarboxylate transporter), member 3 | 64849 | 1,4 | 7,80E-03 |
| IGHG3 | immunoglobulin heavy constant gamma 3 (G3m marker) | 3502 | 1,4 | 1,50E-02 |
| C8orf76 | chromosome 8 open reading frame 76 | 84933 | 1,4 | 5,70E-03 |
| SNORD114-6 | small nucleolar RNA, C/D box 114-6 | 767582 | 1,4 | 1,80E-02 |
| NBPF15 | neuroblastoma breakpoint family, member 15 | 284565 | 1,39 | 2,20E-02 |
| RNF216P1 | ring finger protein 216 pseudogene 1 | 441191 | 1,39 | 1,80E-02 |
| FAM107B | family with sequence similarity 107, member B | 83641 | 1,39 | 6,30E-03 |
| FAM90A10P | putative protein FAM90A10 | 441328 | 1,39 | 3,10E-02 |
| NINJ1 | ninjurin 1 | 4814 | 1,39 | 1,40E-02 |
| STYXL1 | serine/threonine/tyrosine interacting-like 1 | 51657 | 1,39 | 3,10E-03 |
| BEGAIN | brain-enriched guanylate kinase-associated | 57596 | 1,39 | 3,40E-02 |
| TRAJ46 | T cell receptor alpha joining 46 | 28709 | 1,39 | 1,70E-02 |
| LOC100288637 | OTU deubiquitinase 7A pseudogene | 100288637 | 1,38 | 6,90E-03 |
| ANLN | anillin, actin binding protein | 54443 | 1,38 | 7,60E-03 |
| GSTT2 | glutathione S-transferase theta 2 | 2953 | 1,38 | 1,20E-02 |
| HORMAD1 | HORMA domain containing 1 | 84072 | 1,38 | 8,10E-03 |
| NME9 | NME/NM23 family member 9 | 347736 | 1,38 | 3,20E-02 |
| HCAR2 | hydroxycarboxylic acid receptor 2 | 338442 | 1,38 | 1,70E-02 |
| LRRC38 | leucine rich repeat containing 38 | 126755 | 1,38 | 3,40E-02 |
| CYP3A5 | cytochrome P450, family 3, subfamily A, polypeptide 5 | 1577 | 1,38 | 1,10E-02 |
| LOC101929634 | uncharacterized LOC101929634 | 101929634 | 1,37 | 3,00E-03 |
| SCNN1A | sodium channel, non-voltage-gated 1 alpha subunit | 6337 | 1,37 | 1,40E-02 |
| LINC01491 | long intergenic non-protein coding RNA 1491 | 101928442 | 1,37 | 7,90E-03 |
| CYSLTR1 | cysteinyl leukotriene receptor 1 | 10800 | 1,37 | 2,80E-03 |
| C8orf37-AS1 | C8orf37 antisense RNA 1 | 100616530 | 1,37 | 1,90E-02 |
| ADORA2A | adenosine A2a receptor | 135 | 1,37 | 4,20E-03 |
| TRAV8-1 | T cell receptor alpha variable 8-1 | 28685 | 1,37 | 2,00E-03 |
| OR2A1 | olfactory receptor, family 2, subfamily A, member 1 | 346528 | 1,36 | 1,90E-02 |
| TTC9 | tetratricopeptide repeat domain 9 | 23508 | 1,36 | 2,60E-02 |
| LOC101928916 | uncharacterized LOC101928916 | 101928916 | 1,36 | 4,90E-02 |
| IL32 | interleukin 32 | 9235 | 1,36 | 6,40E-03 |
| C8orf31 | chromosome 8 open reading frame 31 | 286122 | 1,36 | 1,10E-02 |
| C9orf173-AS1 | C9orf173 antisense RNA 1 | 100129722 | 1,36 | 1,90E-02 |
| C21orf140 | chromosome 21 open reading frame 140 | 101928147 | 1,35 | 2,80E-03 |
| PCDHB13 | protocadherin beta 13 | 56123 | 1,35 | 4,90E-02 |
| KIAA0513 | KIAA0513 | 9764 | 1,35 | 4,50E-03 |
| UBE2Q2P1 | ubiquitin-conjugating enzyme E2Q family member 2 pseudogene 1 | 388165 | 1,35 | 3,70E-02 |
| LOC647859 | occludin pseudogene | 647859 | 1,35 | 2,30E-02 |
| TBL1X | transducin (beta)-like 1X-linked | 6907 | 1,35 | 9,00E-03 |
| PDIA3 | protein disulfide isomerase family A, member 3 | 2923 | 1,35 | 2,20E-02 |
| LOC100130476 | uncharacterized LOC100130476 | 100130476 | 1,35 | 2,20E-02 |
| MIR670 | microRNA 670 | 100313777 | 1,34 | 2,00E-02 |
| OSTCP1 | oligosaccharyltransferase complex subunit pseudogene 1 | 202459 | 1,34 | 4,20E-03 |
|  |  |  |  |  |
| **Mild intermittent hypoxia exposure - downregulation** | | | | |
| LOC149351 | uncharacterized LOC149351 | 149351 | -1,31 | 2,50E-02 |
| LOC101927002 | uncharacterized LOC101927002 | 101927002 | -1,31 | 3,70E-02 |
| MAS1L | MAS1 proto-oncogene like, G protein-coupled receptor | 116511 | -1,31 | 2,20E-02 |
| FGF22 | fibroblast growth factor 22 | 27006 | -1,31 | 4,10E-03 |
| PUS1 | pseudouridylate synthase 1 | 80324 | -1,31 | 2,70E-02 |
| LINC01140 | long intergenic non-protein coding RNA 1140 | 339524 | -1,31 | 3,80E-02 |
| LOC101928220 | putative IQ motif and ankyrin repeat domain-containing protein LOC642574-like | 101928220 | -1,31 | 1,90E-02 |
| PCOLCE2 | procollagen C-endopeptidase enhancer 2 | 26577 | -1,31 | 2,70E-02 |
| NA | NA | 100996474 | -1,31 | 1,10E-02 |
| MGC16142 | uncharacterized protein MGC16142 | 84849 | -1,31 | 1,30E-02 |
| OR10P1 | olfactory receptor, family 10, subfamily P, member 1 | 121130 | -1,31 | 1,60E-02 |
| LOC101929500 | uncharacterized LOC101929500 | 101929500 | -1,32 | 4,20E-02 |
| C17orf67 | chromosome 17 open reading frame 67 | 339210 | -1,32 | 1,20E-03 |
| RBM12B-AS1 | RBM12B antisense RNA 1 | 55472 | -1,32 | 8,00E-03 |
| MIR1246 | microRNA 1246 | 100302142 | -1,32 | 2,80E-02 |
| CXCL2 | chemokine (C-X-C motif) ligand 2 | 2920 | -1,32 | 4,70E-02 |
| SPACA6P | sperm acrosome associated 6, pseudogene | 147650 | -1,32 | 8,60E-03 |
| MT1M | metallothionein 1M | 4499 | -1,32 | 2,90E-02 |
| EDN1 | endothelin 1 | 1906 | -1,33 | 5,80E-03 |
| GIPC2 | GIPC PDZ domain containing family, member 2 | 54810 | -1,33 | 2,10E-02 |
| ZNF781 | zinc finger protein 781 | 163115 | -1,33 | 6,50E-03 |
| RPL23AP64 | ribosomal protein L23a pseudogene 64 | 649946 | -1,33 | 4,90E-03 |
| SPRR4 | small proline-rich protein 4 | 163778 | -1,33 | 9,20E-03 |
| SURF2 | surfeit 2 | 6835 | -1,33 | 1,10E-02 |
| IFIT3 | interferon-induced protein with tetratricopeptide repeats 3 | 3437 | -1,33 | 1,30E-02 |
| MIR4252 | microRNA 4252 | 100422975 | -1,34 | 3,00E-02 |
| CYP1A1 | cytochrome P450, family 1, subfamily A, polypeptide 1 | 1543 | -1,34 | 2,30E-02 |
| MIR376A2 | microRNA 376a-2 | 664615 | -1,34 | 6,60E-03 |
| FAM109A | family with sequence similarity 109, member A | 144717 | -1,34 | 6,40E-03 |
| BASP1P1 | brain abundant, membrane attached signal protein 1 pseudogene 1 | 646201 | -1,34 | 6,10E-03 |
| PLA2G2A | phospholipase A2, group IIA (platelets, synovial fluid) | 5320 | -1,34 | 1,40E-02 |
| TAS2R50 | taste receptor, type 2, member 50 | 259296 | -1,34 | 2,30E-02 |
| FLJ41278 | uncharacterized LOC400046 | 400046 | -1,35 | 3,50E-02 |
| LOC101928264 | uncharacterized LOC101928264 | 101928264 | -1,35 | 6,80E-03 |
| BTN3A1 | butyrophilin, subfamily 3, member A1 | 11119 | -1,35 | 4,70E-02 |
| PAK3 | p21 protein (Cdc42/Rac)-activated kinase 3 | 5063 | -1,35 | 3,10E-02 |
| LOC101927685 | heat shock transcription factor, X-linked-like | 101927685 | -1,35 | 1,80E-02 |
| PRIM2B | primase, DNA, polypeptide 2 (58kDa) pseudogene | 100996481 | -1,35 | 1,50E-03 |
| CSGALNACT1 | chondroitin sulfate N-acetylgalactosaminyltransferase 1 | 55790 | -1,36 | 6,40E-03 |
| SEC24B-AS1 | SEC24B antisense RNA 1 | 100533182 | -1,36 | 3,90E-02 |
| LINC00421 | long intergenic non-protein coding RNA 421 | 100287114 | -1,36 | 3,70E-03 |
| KIT | v-kit Hardy-Zuckerman 4 feline sarcoma viral oncogene homolog | 3815 | -1,37 | 1,30E-02 |
| ZNF806 | zinc finger protein 806 | 646915 | -1,37 | 2,30E-02 |
| LOC101928948 | uncharacterized LOC101928948 | 101928948 | -1,37 | 2,50E-02 |
| REG3G | regenerating islet-derived 3 gamma | 130120 | -1,37 | 1,90E-02 |
| TAS2R19 | taste receptor, type 2, member 19 | 259294 | -1,37 | 1,50E-02 |
| LLPH | LLP homolog, long-term synaptic facilitation (Aplysia) | 84298 | -1,37 | 8,90E-04 |
| KRTAP13-3 | keratin associated protein 13-3 | 337960 | -1,38 | 4,50E-03 |
| CYP7B1 | cytochrome P450, family 7, subfamily B, polypeptide 1 | 9420 | -1,38 | 5,50E-03 |
| CALCB | calcitonin-related polypeptide beta | 797 | -1,38 | 1,50E-02 |
| IGHV6-1 | immunoglobulin heavy variable 6-1 | 28385 | -1,38 | 1,20E-02 |
| LOC100506127 | putative uncharacterized protein FLJ37770-like | 100506127 | -1,38 | 3,00E-02 |
| TTTY20 | testis-specific transcript, Y-linked 20 (non-protein coding) | 252951 | -1,39 | 1,90E-02 |
| MAGEC2 | melanoma antigen family C, 2 | 51438 | -1,39 | 1,40E-02 |
| SNORA60 | small nucleolar RNA, H/ACA box 60 | 677837 | -1,39 | 4,60E-02 |
| AKR7A2 | aldo-keto reductase family 7, member A2 (aflatoxin aldehyde reductase) | 8574 | -1,39 | 1,40E-02 |
| ZNF37A | zinc finger protein 37A | 7587 | -1,39 | 2,10E-02 |
| CLP1 | cleavage and polyadenylation factor I subunit 1 | 10978 | -1,39 | 2,30E-02 |
| LOC285191 | uncharacterized LOC285191 | 285191 | -1,4 | 2,90E-02 |
| RRP7B | ribosomal RNA processing 7 homolog B (S. cerevisiae) | 91695 | -1,4 | 2,70E-03 |
| ZNF563 | zinc finger protein 563 | 147837 | -1,4 | 2,00E-02 |
| CMC1 | C-x(9)-C motif containing 1 | 152100 | -1,4 | 2,80E-02 |
| NA | NA | 101927261 | -1,4 | 1,30E-02 |
| TRAV13-2 | T cell receptor alpha variable 13-2 | 28670 | -1,4 | 3,90E-03 |
| KCNAB1 | potassium voltage-gated channel, shaker-related subfamily, beta member 1 | 7881 | -1,4 | 1,80E-02 |
| NA | NA | 101927091 | -1,41 | 4,40E-02 |
| CMIP | c-Maf inducing protein | 80790 | -1,42 | 1,30E-02 |
| SPRNP1 | shadow of prion protein homolog (zebrafish) pseudogene 1 | 399833 | -1,42 | 4,10E-02 |
| LINC00882 | long intergenic non-protein coding RNA 882 | 100302640 | -1,42 | 1,10E-03 |
| PTENP1 | phosphatase and tensin homolog pseudogene 1 (functional) | 11191 | -1,43 | 8,00E-05 |
| MIR711 | microRNA 711 | 100313843 | -1,43 | 4,50E-03 |
| RPL12 | ribosomal protein L12 | 6136 | -1,43 | 1,30E-03 |
| OR4C46 | olfactory receptor, family 4, subfamily C, member 46 | 119749 | -1,44 | 1,60E-04 |
| NA | NA | 101929686 | -1,44 | 4,80E-02 |
| NBPF18P | neuroblastoma breakpoint family, member 18, pseudogene | 441908 | -1,45 | 1,40E-02 |
| MIR30D | microRNA 30d | 407033 | -1,45 | 3,20E-02 |
| LCE1E | late cornified envelope 1E | 353135 | -1,45 | 3,30E-03 |
| SNORD113-8 | small nucleolar RNA, C/D box 113-8 | 767568 | -1,45 | 1,80E-02 |
| LOC100506470 | uncharacterized LOC100506470 | 100506470 | -1,46 | 7,20E-03 |
| ZNF304 | zinc finger protein 304 | 57343 | -1,47 | 2,50E-03 |
| FAM182A | family with sequence similarity 182, member A | 284800 | -1,47 | 3,00E-02 |
| KRT19P2 | keratin 19 pseudogene 2 | 160313 | -1,47 | 4,20E-02 |
| CDC14C | cell division cycle 14C | 168448 | -1,47 | 2,80E-03 |
| PRG4 | proteoglycan 4 | 10216 | -1,48 | 4,20E-02 |
| MILR1 | mast cell immunoglobulin-like receptor 1 | 284021 | -1,49 | 8,10E-03 |
| APOC4-APOC2 | APOC4-APOC2 readthrough (NMD candidate) | 100533990 | -1,5 | 3,80E-02 |
| LOC101928269 | uncharacterized LOC101928269 | 101928269 | -1,52 | 3,00E-02 |
| CA14 | carbonic anhydrase XIV | 23632 | -1,53 | 2,90E-02 |
| MIR1273F | microRNA 1273f | 100616156 | -1,53 | 3,70E-02 |
| LOC101929251 | uncharacterized LOC101929251 | 101929251 | -1,54 | 6,50E-03 |
| MIR3928 | microRNA 3928 | 100500901 | -1,55 | 2,20E-02 |
| LINC01489 | long intergenic non-protein coding RNA 1489 | 101928340 | -1,55 | 9,40E-03 |
| GEMIN2 | gem (nuclear organelle) associated protein 2 | 8487 | -1,56 | 2,30E-04 |
| LOC439933 | uncharacterized LOC439933 | 439933 | -1,57 | 1,30E-03 |
| FOLH1 | folate hydrolase (prostate-specific membrane antigen) 1 | 2346 | -1,58 | 1,80E-02 |
| SNORD114-4 | small nucleolar RNA, C/D box 114-4 | 767580 | -1,6 | 2,50E-02 |
| OR5P2 | olfactory receptor, family 5, subfamily P, member 2 | 120065 | -1,64 | 1,40E-02 |
| MAGOHB | mago-nashi homolog B (Drosophila) | 55110 | -1,65 | 4,80E-04 |
| LOC101928722 | flocculation protein FLO11-like | 101928722 | -1,7 | 3,80E-02 |
| TRBJ2-6 | T cell receptor beta joining 2-6 | 28623 | -1,7 | 1,10E-02 |

### **Supplementary Table 3**. Primer sequences used for gene expression analysis in primary human adipocytes.

|  | | Sequence |
| --- | --- | --- |
| IL-6 | Forward | AAATTCGGTACATCCTCGACGG |
|  | Reverse | GGAAGGTTCAGGTTGTTTTCTGC |
| MCP-1 | Forward | CCCCAGTCACCTGCTGTTAT |
|  | Reverse | TCCTGAACCCACTTCTGCTT |
| PAI-1 | Forward | TCGTCCAGCGGGATCTGAA |
|  | Reverse | GCCGTTGAAGTAGAGGGCATT |
| Leptin | Forward | GCTGTGCCCATCCAAAAAGTCC |
|  | Reverse | CCCAGGAATGAAGTCCAAACCG |
| GLUT1 | Forward | TTGCAGGCTTCTCCAACTGGAC |
|  | Reverse | CAGAACCAGGAGCACAGTGAAG |
| HSL | Forward | GCGGATCACACAGAACCTGGAC |
|  | Reverse | AGCAGGCGGCTTACCCTCAC |
| VEGFA | Forward | TTGCCTTGCTGCTCTACCTCCA |
|  | Reverse | GATGGCAGTAGCTGCGCTGATA |
| ACAB | Forward | GCAAGAACGTGTGGGGTTACT |
|  | Reverse | TCGCCTCGGATGGACAGTT |
| SCD1 | Forward | CCTGGTTTCACTTGGAGCTGTG |
|  | Reverse | TGTGGTGAAGTTGATGTGCCAGC |
| FASN | Forward | CCGAGACACTCGTGGGCTA |
|  | Reverse | CTTCAGCAGGACATTGATGCC |
| BNIP3 | Forward | ATCAAAAGGTGCTGGTGGAG |
|  | Reverse | ACCCTCAGCATGAGGAACAC |
| CA9 | Forward | CATCCTAGCCCTGGTTTTTGG |
|  | Reverse | GCTCACACCCCCTTTGGTT |
| GLUT1* | Forward | GATTGGCTCCTTCTCTGTGG |
|  | Reverse | TCAAAGGACTTGCCCAGTTT |
| VEGFA* | Forward | GACTCCGGCGGAAGCAT |
|  | Reverse | TCCGGGCTCGGTGATTTA |
| 18S | Forward | AGTTAGCATGCCAGAGTCTCG |
|  | Reverse | TGCATGGCCGTTCTTAGTTG |
| RPL13A | Forward | CCTGGAGGAGAAGAGGAAAGAGA |
|  | Reverse | TTGAGGACCTCTGTGTATTTGTCAA |

*, differential primer sequence used in myotube experiments.
